# Chloride homeostasis dysfunction drives hyperactivation of corticotropin-releasing factor-expressing neurons in the amygdala in stress-induced hypertension

**DOI:** 10.1172/JCI195536

**Published:** 2026-03-16

**Authors:** Hongyu Ma, Ying Zhang, Xinqi Guo, Qiyue Zhao, Peiyun Yang, Yan Liu, Yue Guan, Yan Wei, Huijie Ma

**Affiliations:** 1 Department of Physiology, Hebei Medical University, Shijiazhuang, Hebei, China.; 2 The Key Laboratory of Neural and Vascular Biology, Ministry of Education, Hebei Medical University, Shijiazhuang, China.; 3 Departments of Endocrinology, The Third Hospital of Hebei Medical University, Shijiazhuang, Hebei, China.; 4 Key Laboratory of Medical Electrophysiology, Ministry of Education & Medical Electrophysiological Key Laboratory of Sichuan Province, Institute of Cardiovascular Research, Southwest Medical University, Luzhou, China.; 5 Hebei Key Laboratory of Brain Science and Brain-Inspired Intelligence, Shijiazhuang, Hebei, China.

**Keywords:** Cell biology, Neuroscience, Chloride channels, Hypertension, Synapses

## Abstract

Stress promotes the progression from borderline hypertension to sustained hypertension, but the mechanism remains unclear. We investigated the role of corticotropin-releasing factor (CRF)-expressing neurons in the central nucleus of amygdala (CeA) on arterial blood pressure (ABP) and sympathetic activity of borderline hypertensive rats (BHRs) subjected to chronic unpredictable mild stress (CUMS). CUMS induced sustained hypertension, and led to increased delta-FosB expression as well as enhanced spontaneous and evoked firing of CeA CRF-expressing neurons in BHRs. Furthermore, optogenetic activation of CeA CRF-expressing neurons significantly increased the sympathetic outflow and ABP in BHRs. Impaired GABAergic inhibition, a depolarizing shift of GABA reversal potential (*E*_GABA_), disrupted chloride homeostasis and increased NKCC1 expression were observed in CeA CRF-expressing neurons in BHRs subjected to CUMS. NKCC1 inhibition with bumetanide restored GABAergic inhibition and chloride homeostasis, normalized neuronal excitability, leading to reduced sympathetic vasomotor tone in CUMS BHRs. These results indicate that NKCC1-mediated disruption of chloride homeostasis in CeA CRF-expressing neurons contributes to elevated sympathetic activity and hypertension under chronic stress. These findings enhance our understanding of the neuronal and molecular mechanisms underlying stress-induced hypertension and reveal potential targets for its prevention and treatment.

## Abstract 2

NKCC1-mediated disruption of chloride homeostasis in CeA CRF-expressing neurons contributes to elevated sympathetic activity and hypertension under chronic stress

## Graphical abstract

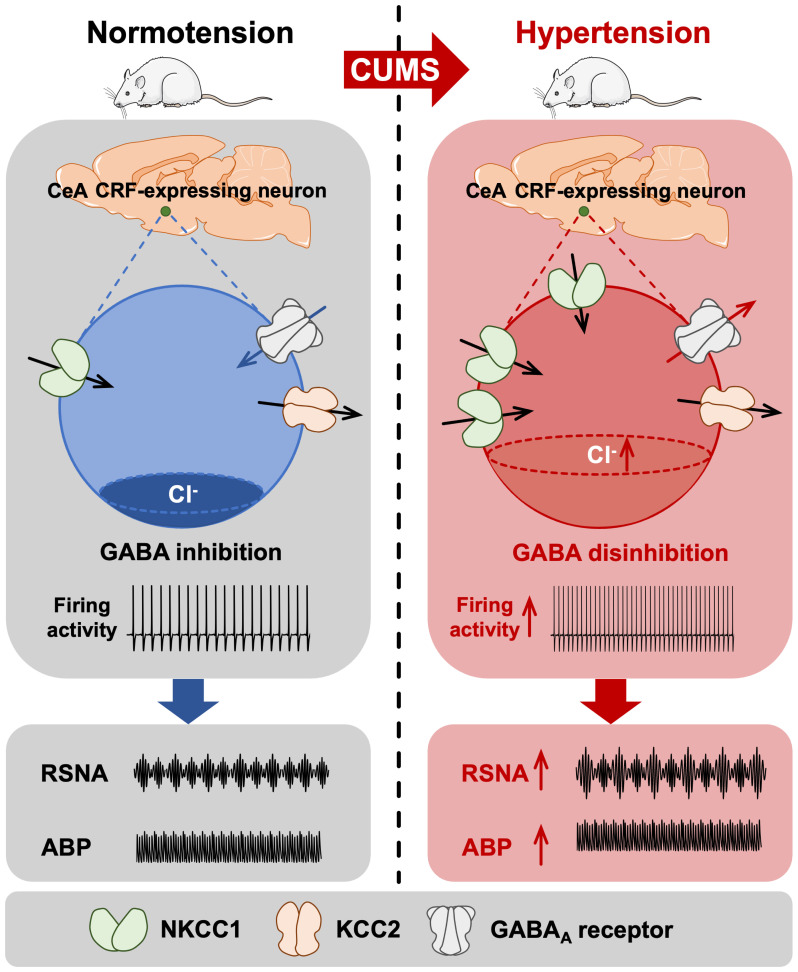

## Introduction

Hypertension affects over 1 billion adults worldwide and remains a leading cause of cardiovascular morbidity and mortality, contributing to severe complications such as stroke, heart failure, and chronic kidney disease (1, 2). Its development is influenced by a complex interplay of genetic predisposition, environmental factors, unhealthy lifestyles, and cardiovascular system dysfunction (3). Among these, chronic stress has been increasingly recognized as a critical risk factor for hypertension (4, 5). Individuals with borderline hypertension, particularly those with a genetic predisposition, are more susceptible to developing sustained hypertension when exposed to repeated chronic stress (6). However, the precise mechanisms underlying chronic stress-induced hypertension remain inadequately understood. The borderline hypertensive rat (BHR), a first generation offspring of the spontaneously hypertensive rat (SHR) and the normotensive Wistar-Kyoto rat (WKY), develops sustained hypertension when exposed to chronic stress or a long-term high-salt diet (7, 8). BHR serves as an established experimental model for investigating hypertension that arises from the interplay between environmental and genetic factors (9, 10).

Chronic stress activates corticotropin-releasing factor (CRF)-expressing neurons in the central nucleus of the amygdala (CeA), a key extrahypothalamic region involved in regulating cardiovascular function during psychological stress, fear, and anxiety (11, 12). The excitability of CRF-expressing neurons is regulated by glutamatergic excitatory input (13) and γ-aminobutyric acid (GABA) inhibitory input (14). GABA, the primary inhibitory neurotransmitter in the mature brain, reduces neuronal excitability by hyperpolarizing membrane potentials through tonic GABA_A_ receptor-mediated chloride ion (Cl^–^) influx (15). Chronic stress has been found to impair GABAergic regulation of CRF-expressing neurons in the amygdala by suppressing tonic GABA_A_ receptor currents (16). However, the role of GABA_A_ receptors in CeA CRF-expressing neurons in modulating sympathetic outflow, as well as their contribution to the development of chronic stress-induced hypertension, remains poorly understood.

The impairment of GABA_A_ receptors is often associated with altered expression of chloride transporters, particularly the Na^+^-K^+^-2Cl^–^ cotransporter-1 (NKCC1) and the K^+^-Cl^–^ cotransporter-2 (KCC2), which are essential for maintaining chloride homeostasis and neuronal excitability in the central nervous system (17, 18). Dysregulated expression of NKCC1 and KCC2 has been implicated in various neurological and neuropsychiatric disorders, including Alzheimer’s disease, Parkinson’s disease, schizophrenia, and neuropathic hypertension (19, 20). Recent studies have highlighted the critical role of chloride homeostasis in stress-related disorders (21). In this study, we demonstrate that dysfunction in NKCC1-mediated GABAergic inhibition disrupts chloride homeostasis in CeA CRF-expressing neurons, leading to increased sympathetic outflow and elevated arterial blood pressure (ABP) in chronic stress-induced hypertension.

## Results

### CUMS leads to persistent hypertension and increases activation of CeA CRF-expressing neurons in BHRs.

**Figure 1:**
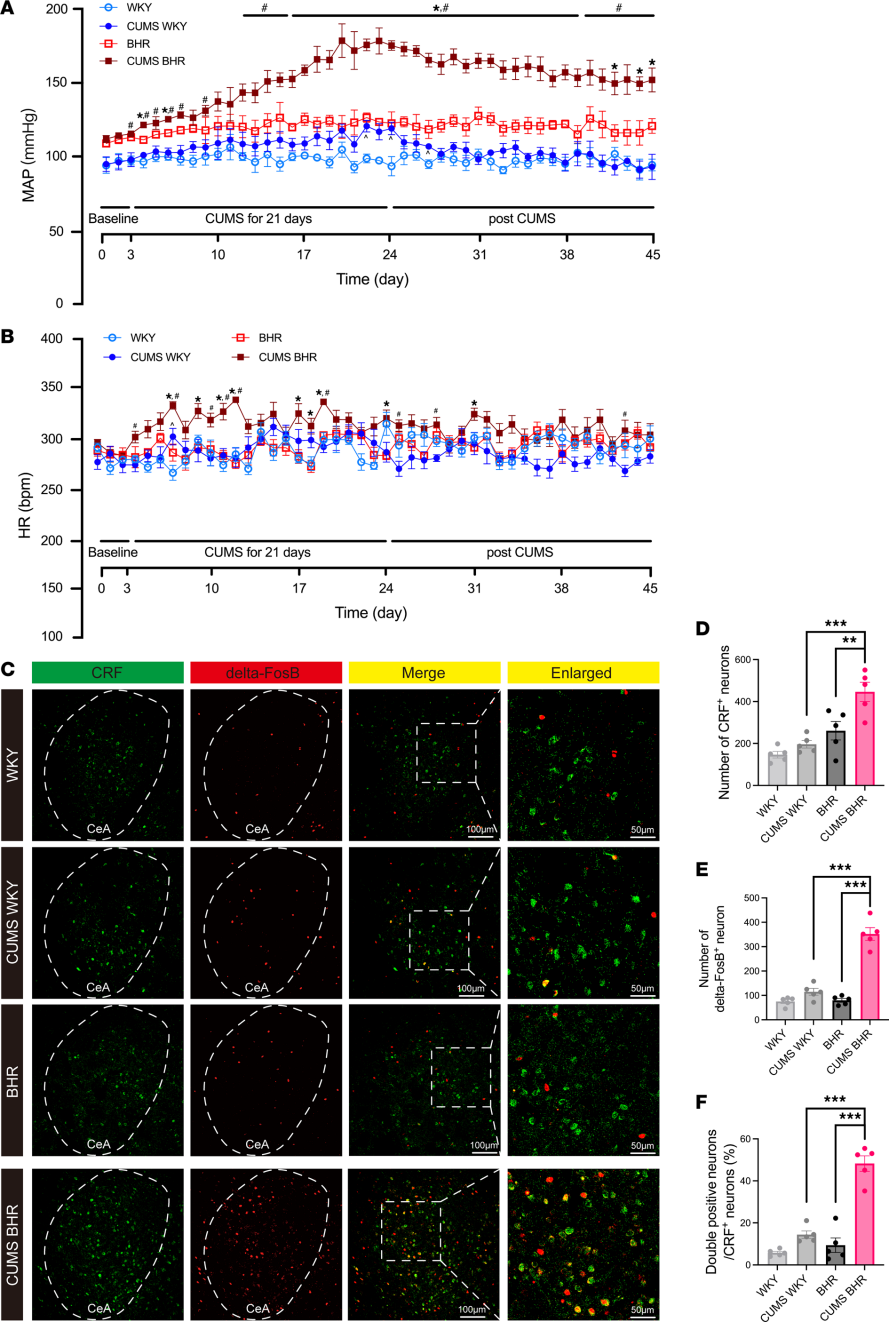
CUMS induces sustained hypertension and increased activation of CeA CRF-expressing neurons in BHRs. (**A** and **B**) Changes in MAP (**A**) and HR (**B**) measured by radiotelemetry before, during and after CUMS in WKY rats, BHRs and age-matched unstressed WKY rats and BHRs (*n* = 6 rats in each group). Data are expressed as means ± SEM. *P < 0.05 for CUMS BHR versus unstressed BHRs, ^#^*P* < 0.05 for CUMS BHR versus CUMS WKY, ^*P* < 0.05 for CUMS WKY versus unstressed WKY. Repeated measures of 2-way ANOVA with Tukey’s post hoc test. (**C**) Representative immunofluorescence images showing CRF immunopositive neurons (green), delta-FosB immunopositive neurons (red), and CRF and delta-FosB double-positive neurons in the CeA of WKY, CUMS WKY, BHR, and CUMS BHR group of rats (*n* = 5 rats in each group). (**D** and **E**) Summary data showing the numbers of CRF-positive neurons (**D**) and delta-FosB positive neurons (**E**) in the CeA of these 4 groups. (**F**) The percentage of CRF and delta-FosB double-positive neurons in the CeA of these 4 groups (*n* = 5 rats in each group). Data are expressed as means ± SEM. **P < 0.01, ***P < 0.001. One-way ANOVA with Tukey’s multiple comparison tests.

We conducted a 21-day CUMS on BHRs and age-matched WKY rats. Before the CUMS, we recorded the baseline of mean arterial blood pressure (MAP) and heart rate (HR) for 3 days. The baseline MAP of BHRs was significantly higher than that in WKY rats (*P* = 0.0290, *t*_10_ = 2.548). CUMS-treatment elevated the MAP of both WKY rats and BHRs. However, the CUMS-induced ABP elevations were significantly higher and more sustained in BHRs than in WKY rats. Moreover, the MAP in CUMS BHRs remained elevated for 21 days, while in WKY rats, it returned to normal shortly after CUMS ended (*n* = 6 rats, Figure 1A). BHRs had a baseline HR similar to WKY rats (*P* = 0.7072, *t*_10_ = 0.3865). CUMS significantly increased HR in BHRs but did not affect HR in WKY rats (*n* = 6 rats, Figure 1B). We also examined the effect of CUMS on serum corticosterone (CORT) levels. Serum CORT level of CUMS BHRs was significantly increased compared to unstressed BHRs (*P* = 0.0110, *F*_3,36_ = 5.155, *n* = 10 rats per group, Supplemental Figure 1; supplemental material available online with this article; https://doi.org/10.1172/JCI195536DS1). However, no significant difference was observed between WKY and CUMS-exposed WKY rats. Since CUMS had little effect on normotensive WKY rats, therefore we mainly focused our subsequent studies on the mechanisms underlying CUMS-induced changes in BHRs.

It is well known that stress increases the expression level of CRF in the paraventricular nucleus (PVN), CeA, or hippocampus (22). However, the changes in the excitability of CRF-expressing neurons in the CeA in chronic stress-induced hypertension are still unclear. Here, we determined the delta-FosB immunoactivity of CRF-expressing neurons using immunofluorescence staining (*n* = 5 rats, Figure 1C). We found that the numbers of CRF-positive neurons (*P* = 0.0066, *F*_3,16_ = 15.00) and delta-FosB-positive neurons (*P* < 0.0001, *F*_3,16_ = 70.73) were significantly increased in CUMS BHRs (Figure 1, D and E). Meanwhile, the percentage of delta-FosB immunoactivity in CRF-expressing neurons (*P* < 0.0001, *F*_3,16_ = 52.47) was much higher in CUMS BHRs (Figure 1F). These results suggested that CUMS induces a sustained hypertension and increased activity of CRF-expressing neurons in BHRs.

### CUMS induces hyperexcitability of CeA CRF-expressing neurons in BHRs.

**Figure 2:**
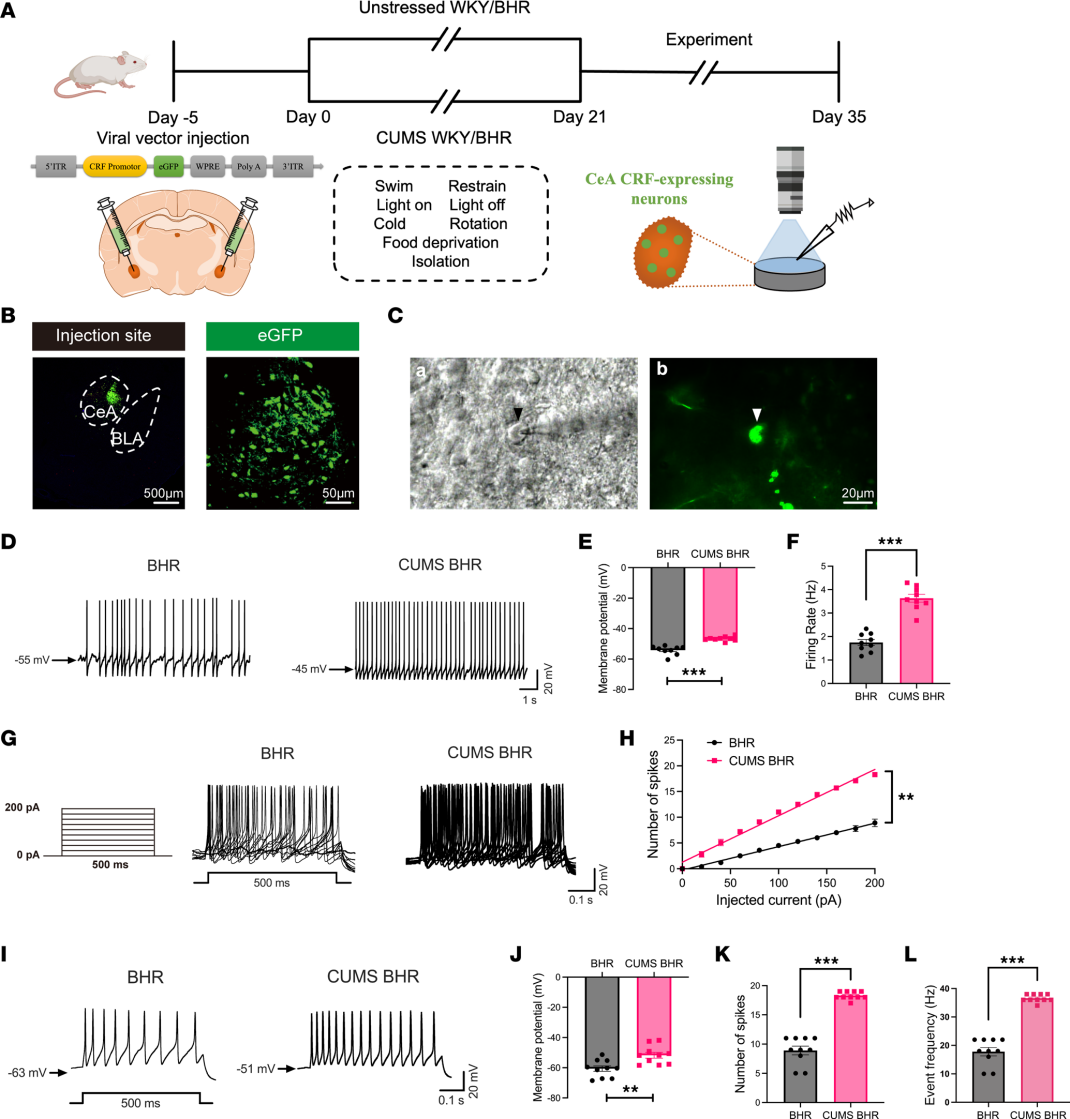
CUMS induces hyperexcitability of CeA CRF-expressing neurons in BHRs. (**A**) Experimental flow diagram and schematic illustrate the microinjection of AAV vector with CRF-promoter driven eGFP into the CeA of WKY, CUMS WKY, BHR and CUMS BHR groups of rats, and subsequent patch-clamp recordings of labeled CRF-expressing neurons in the CeA from these four groups of rats. (**B**) Representative images show the viral vector injection site and eGFP-labeled neurons (green) within the CeA. (**C**) An eGFP-labeled CRF-expressing neuron with an attached glass recording microelectrode viewed with infrared differential interference contrast optics (a) and fluorescence illumination (b) in a brain slice from BHRs. (**D**) Raw traces show spontaneous firing activity of CRF-expressing neurons in the CeA of BHR and CUMS BHR. (**E** and **F**) Summary data show the membrane potential (**E**) and firing rate (**F**) of spontaneous firing activity of CRF-expressing neurons in the CeA of BHR and CUMS BHR (*n* = 9 neurons in each group). (**G**) Protocol for current injection (0–200 pA in 20 pA increments, 500 ms duration) and representative traces of current evoked action potentials of CRF-expressing neurons in the CeA of BHR and CUMS BHR. (**H**) Summary data show the number of spikes in currents evoked firing activity of CRF-expressing neurons in the CeA of BHR and CUMS BHR (*n* = 10 neurons in each group). (**I**) Representative traces show action potentials evoked by +200 pA current injection of CRF-expressing neurons in the CeA of BHR and CUMS BHR. (**J**–**L**) Summary data show the membrane potential (**J**), number of spikes (**K**) and event frequency (**L**) of action potential evoked by +200 pA current injection of CRF-expressing neurons in the CeA of BHR and CUMS BHR (*n* = 10 neurons in each group). CeA, central nucleus of amygdala. BLA, basolateral nucleus of amygdala. Data are expressed as means ± SEM. **P < 0.01, ***P < 0.001. Two-tailed Student’s *t*-test.

Next, we determined the excitability of CRF-expressing neurons in the CeA of BHRs and CUMS BHRs. To selectively label CRF-expressing neurons, we microinjected a rat CRF promoter-driven viral vector into the CeA. After 21 days, we measured the spontaneous firing activity and current-evoked firing activity of CeA CRF-expressing neurons in these four groups (Figure 2A). Perforated patch-clamp recordings were used to measure the spontaneous firing activity of eGFP-labeled CeA CRF-expressing neurons (Figure 2, B and C). The results showed that CUMS BHRs exhibited a depolarized membrane potential (*P* < 0.0001, *t*_16_ = 6.999) and an increased spontaneous firing rate (*P* < 0.0001, *t*_16_ = 8.956) in CeA CRF-expressing neurons compared with BHRs (*n* = 9 neurons, Figure 2, D–F). However, CUMS did not affect the membrane potential or spontaneous firing activity of CeA CRF-expressing neurons in WKY rats (Supplemental Figure 2, A–C). Using whole-cell current-clamp recordings, we measured the step currents-evoked firing activity. The number of spikes of both BHRs and CUMS BHRs were significantly increased with the injection currents increasing. Moreover, at every injection current, the number of spikes of CRF-expressing neurons in CUMS BHRs was much higher than that in unstressed BHRs (*n* = 10 neurons, *P* < 0.01, Figure 2, G and H). To further compare neuronal excitability, we selected +200 pA current-evoked firing and analyzed the membrane potential, number of spikes, and event frequency in CeA CRF-expressing neurons between BHRs and CUMS BHRs (Figure 2I). The membrane potential (*P* = 0.0035, *t*_18_ = 3.362) was depolarized, and the number of spikes (*P* < 0.0001, *t*_18_ = 12.25) and event frequency (*P* < 0.0001, *t*_18_ = 12.25) were increased in CeA CRF-expressing neurons of CUMS BHRs compared to BHRs (*n* = 10 neurons, Figure 2, J–L). However, the membrane potential and evoked firing frequency of CeA CRF-expressing neurons did not differ between unstressed WKY rats and CUMS WKY rats (Supplemental Figure 2, D–G). Taken together, these findings indicate that CUMS potentiates the excitability of CRF-expressing neurons in the CeA of BHRs.

### Manipulating the excitability of CeA CRF-expressing neurons directly affects the sympathetic outflow and ABP in BHRs.

**Figure 3:**
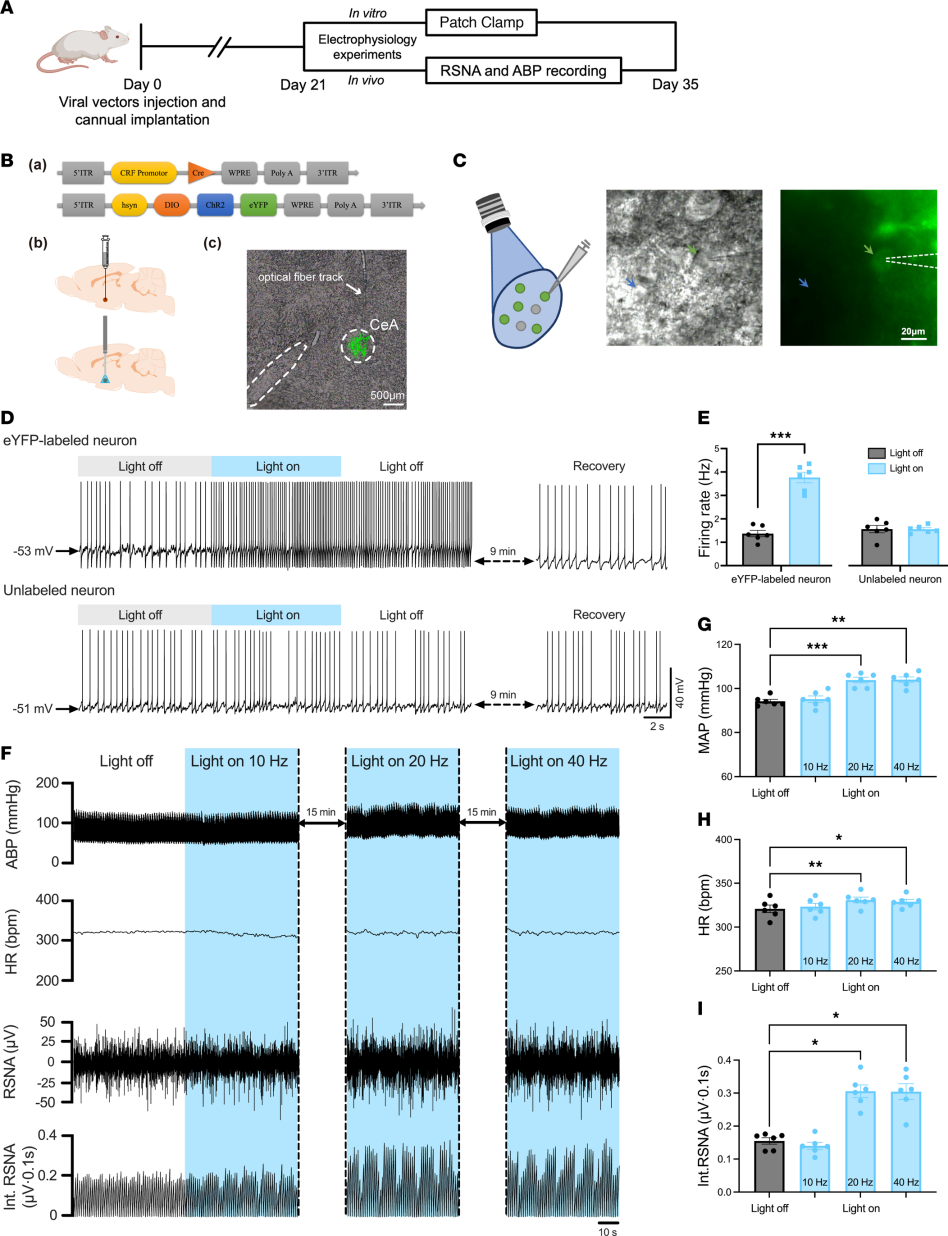
Optogenetic activation of CeA CRF-expressing neurons increases the sympathetic outflow and ABP in BHRs. (**A**) Experimental flow diagram shows the optogenetic viral vector injection to the CeA of rats and subsequent in vitro patch clamp recording and in vivo ABP and RSNA recording. (**B**) Schematic diagram shows the constructs of AAV vectors (a), injection of AAV vectors and implantation of optical fiber (b), and ChR2-eYFP expression within the CeA (c). (**C**) CeA CRF-expressing neurons were activated by blue illumination (473 nm, energy 5 mW, duration 7 seconds and delay 3 seconds in 10 seconds, total 60 seconds). The green arrow indicates an eYFP-labeled neuron with an attached recording electrode, and the blue arrow indicates an unlabeled control neuron. (**D** and **E**) Original traces (**D**) and summary data (**E**) show the effect of blue light illumination on the spontaneous firing activity of both eYFP labeled and unlabeled neurons in the CeA of BHRs (*n* = 6 neurons in each group). Two-tailed Student’s *t*-test. (**F**) Original recording traces show the effects of blue light stimulation of CeA CRF-expressing neurons at different frequencies (10 Hz, 20 Hz, 40 Hz) on ABP, HR, RSNA, and Int. RSNA of BHRs. (**G**–**I**) Summary data show changes of MAP (**G**), HR (**H**), Int. RSNA (**I**) in response to optostimulation of CeA CRF-expressing neurons in BHRs (*n* = 6 rats in each group). One-way ANOVA with Tukey’s multiple comparison tests. CeA, central nucleus of amygdala. Data are expressed as means ± SEM. *P < 0.05, **P < 0.01, ***P < 0.001.

We demonstrated that CUMS increased the ABP and potentiated the excitability of CeA CRF-expressing neurons in BHRs in the above experiments. However, it is not clear whether there is a direct link between the elevated ABP and potentiated excitability of CeA CRF-expressing neurons in CUMS BHRs. Thus, we microinjected a mixture of AAV-CRF-Cre and AAV-hsyn-DIO-ChR2-eYFP into the CeA to selectively express ChR2-eYFP in CeA CRF-expressing neurons in BHRs (Figure 3, A–C). We found the blue light significantly increased the firing rate of eYFP-labeled CeA CRF-expressing neurons (*n* = 6 neurons, *P* = 0.0007, *t*_5_ = 7.507, Figure 3, D and E), whereas it had no effect on unlabeled neurons (*n* = 6 neurons, *P* = 0.9677, *t*_5_ = 0.0425, Figure 3, D and E) in BHRs.

Next, we determined whether selectively activating CeA CRF-expressing neurons could enhance sympathetic outflow and increase the ABP. Optogenetic stimulation of CeA CRF-expressing neurons at different frequencies was applied to assess changes in ABP, HR, and renal sympathetic nerve activity (RSNA) in anesthetized BHRs. We found that blue light photostimulation at 20 Hz or 40 Hz significantly increased MAP, HR and RSNA in BHRs expressing ChR2 in the CeA (*n* = 6 rats, Figure 3, F–I). Conversely, we delivered the chemogenetic inhibitor hM4Di-mCherry into CeA CRF-expressing neurons and used the designer compound clozapine N-oxide (CNO) to selectively inhibit these neurons (Supplemental Figure 3, A–C). Systemic administration of CNO for 28 days significantly reversed CUMS-induced hypertension in BHRs (Supplemental Figure 3, D and E). These data demonstrate that CeA CRF-expressing neurons play a key role in CUMS-induced hypertension.

### CUMS decreases GABAergic inhibition and causes depolarizing shift of E_GABA_ in CeA CRF-expressing neurons in BHRs.

**Figure 4:**
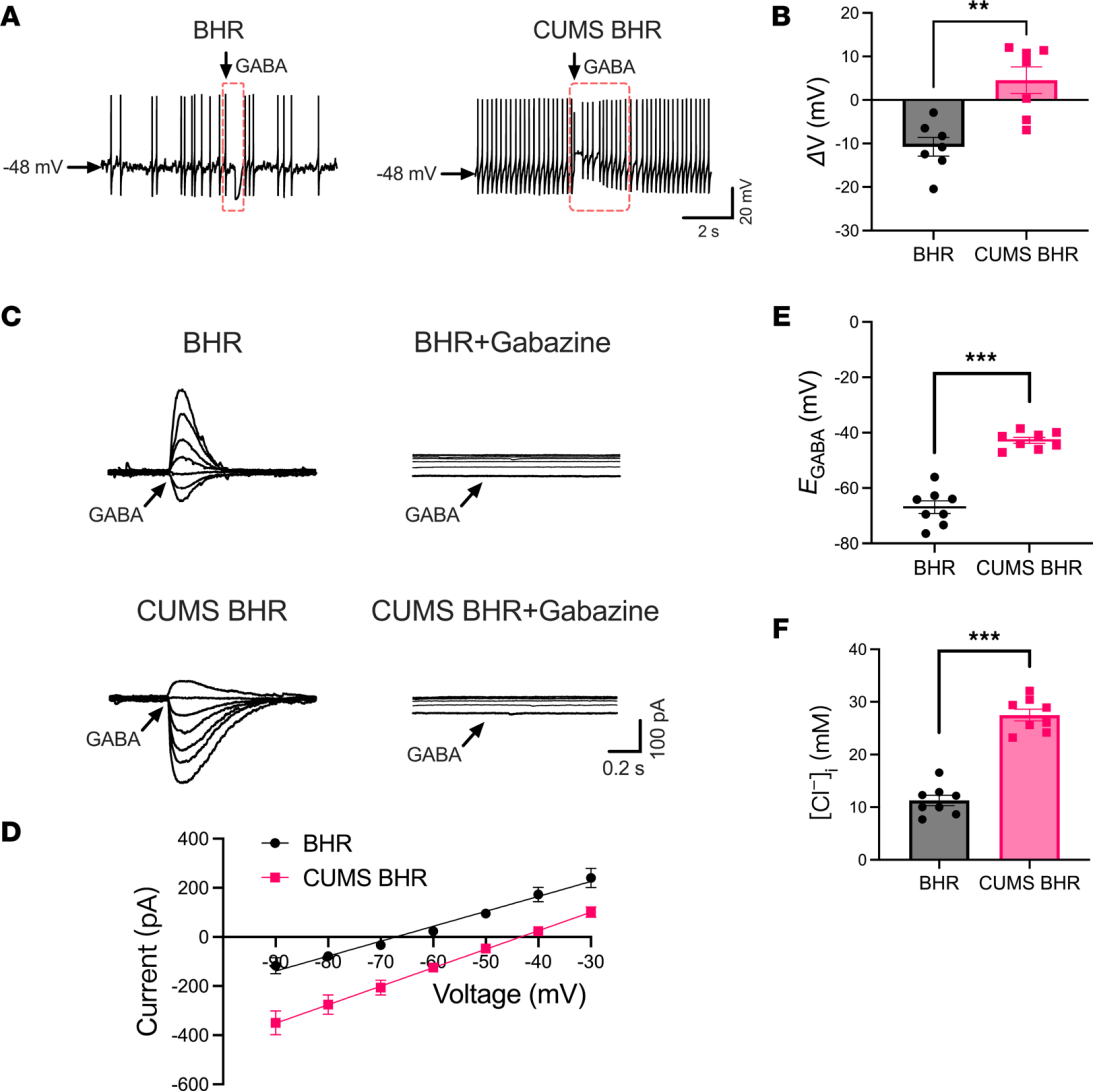
CUMS decreases GABAergic inhibition and causes depolarizing shift of *E*_GABA_ in CeA CRF-expressing neurons in BHRs. (**A**) Original recordings show changes in membrane potential induced by puff application GABA (300 μM) to eGFP-labeled CeA CRF-expressing neurons of BHRs and CUMS BHRs. (**B**) Mean changes in membrane potential (Δ*V*) induced by puff application of GABA to eGFP-labeled CeA neurons in BHR and CUMS BHR (*n* = 7 neurons in each group). (**C** and **D**) Representative perforated patch recordings of GABA-induced currents at a series of membrane potentials ranging from –90 to –30 mV (**C**) and *I-V* plots (**D**) show the *E*_GABA_ of CeA CRF-expressing neurons from BHR and CUMS BHR. Note that GABA-induced currents of eGFP-labeled CeA neurons from both BHR and CUMS BHR were completely blocked by GABA_A_ receptor antagonist gabazine (20 μM). (**E** and **F**) Summary data show changes in *E*_GABA_ (**E**) and derived [Cl^–^]_i_ (**F**) in CeA CRF-expressing neurons of BHR and CUMS BHR (*n* = 8 neurons in each group). The arrows show the timepoint of puff application of GABA. Data are expressed as means ± SEM. **P < 0.01, ***P < 0.001. Two-tailed Student’s *t*-test.

GABAergic inhibition is essential for regulating neuronal excitability (21, 23). We first determined GABA and GABA_A_ receptor levels in the CeA, and found no significant differences among WKY, CUMS WKY, BHR, and CUMS BHR groups (Supplemental Figure 4, A and B). To evaluate the responsiveness of CeA CRF-expressing neurons to GABA, we puffed GABA during spontaneous firing activity in both BHRs and CUMS BHRs. In BHRs, puff application of GABA hyperpolarized the membrane potential of CeA CRF-expressing neurons. In contrast, puff GABA paradoxically depolarized the membrane potential of these neurons in CUMS BHRs (*n* = 7 neurons, *P* = 0.0015, *t*_12_ = 4.107, Figure 4, A and B). We then performed gramicidin-perforated patch-clamp recording of CeA CRF-expressing neurons to determine the GABA reversal potential (*E*_GABA_) and [Cl^–^]_i_. CUMS BHRs showed a significant depolarizing shift (+21.5 mV) in *E*_GABA_ of CeA CRF-expressing neurons compared to unstressed BHRs (*n* = 8 neurons, *P* < 0.0001, *t*_14_ = 9.493, Figure 4, C–E). Meanwhile, the [Cl^–^]_i_ of CeA CRF-expressing neurons, derived from *E*_GABA_ using the Nernst equation, was significantly higher in CUMS BHRs compared to BHRs (*n* = 8 neurons, *P* < 0.0001, *t*_14_ =10.80, Figure 4F). However, CUMS did not affect the *E*_GABA_ and [Cl^–^]_i_ of CeA CRF-expressing neurons in WKY rats (Supplemental Figure 5, A–D). These results indicate a reduction in GABAergic inhibition and a depolarizing shift of *E*_GABA_ in CeA CRF-expressing neurons following CUMS in BHRs.

### CUMS upregulates NKCC1 without affecting KCC2 in the CeA of BHRs.

**Figure 5:**
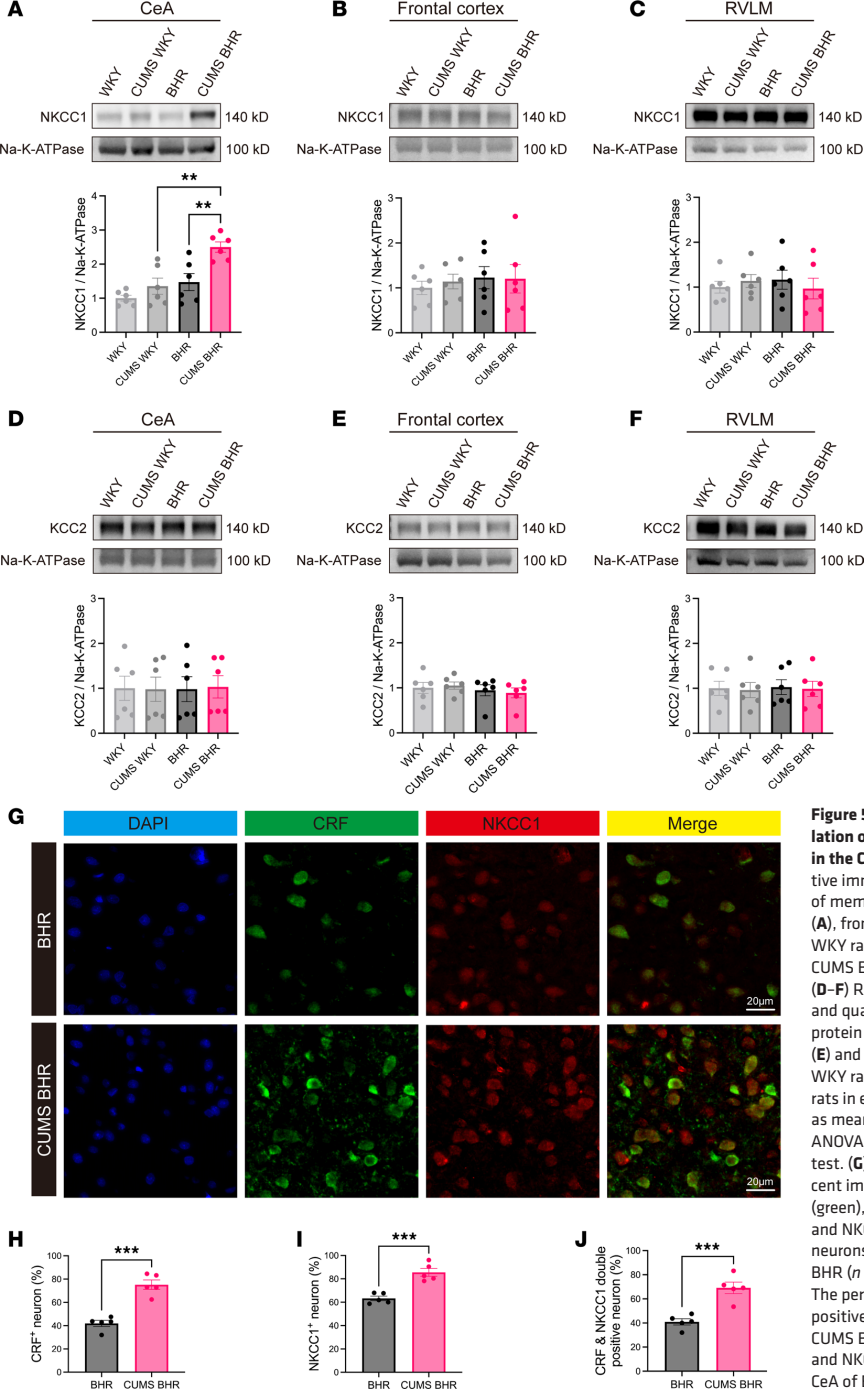
CUMS results in the up-regulation of NKCC1 without affecting KCC2 in the CeA of BHRs. (**A**–**C**) Representative immunoblots and quantification of membrane NKCC1 protein in the CeA (**A**), frontal cortex (**B**) and RVLM (**C**) of WKY rats, CUMS WKY rats, BHRs and CUMS BHRs (*n* = 6 rats in each group). (**D**–**F**) Representative immunoblots and quantification of membrane KCC2 protein in the CeA (**D**), frontal cortex (**E**) and RVLM (**F**) of WKY rats, CUMS WKY rats, BHRs and CUMS BHRs (*n* = 6 rats in each group). Data are expressed as means ± SEM. **P < 0.01. One-way ANOVA followed by Tukey’s post hoc test. (**G**) Representative immunofluorescent images show CRF-positive neurons (green), NKCC1-positive neurons (red), and NKCC1 and CRF double-labeled neurons in the CeA of BHR and CUMS BHR (*n* = 5 rats in each group). (**H** and **I**) The percentage of CRF (**H**) or NKCC1 (**I**) positive neurons in the CeA of BHR and CUMS BHR. (**J**) The percentage of CRF and NKCC1 double positive neurons in the CeA of BHR and CUMS BHR. CeA, central nucleus of amygdala. RVLM, rostral ventrolateral medulla. Data are expressed as means ± SEM. ***P < 0.001. Two-tailed Student’s *t*-test.

The [Cl^–^]_i_ of neurons in the central nervous system is mainly maintained by NKCC1 and KCC2 (24). To determine whether NKCC1 or KCC2 was altered in the CeA of CUMS BHRs, we collected CeA, frontal cortex (FC) and RVLM tissues from WKY rats, CUMS WKY rats, BHRs and CUMS BHRs to measure the membrane protein levels of NKCC1 and KCC2 (Figure 5, A–F). Notably, NKCC1 protein expression in the CeA was much higher in CUMS BHRs than that in unstressed BHRs (*P* = 0.0061, *F*_3,20_ = 11.15, Figure 5A), while KCC2 protein levels in the CeA did not differ significantly between the two groups (Figure 5D). Besides, there was no significant differences in the membrane protein expression levels of NKCC1 and KCC2 in the CeA between CUMS and unstressed WKY rats (Figure 5, A–D). The protein expression levels of NKCC1 or KCC2 showed no obvious differences among all the four groups in the control brain regions, including the FC and RVLM (Figure 5, B, C, E, and F).

To assess the co-localization of CRF-expressing neurons and NKCC1, we employed immunofluorescence double staining to label CRF-positive and NKCC1-positive neurons in the CeA (*n* = 5 rats in each group, Figure 5G). The results showed that the percentages of CRF-positive neurons and NKCC1 positive neurons were increased in the CeA (Figure 5, H and I). Additionally, CUMS significantly increased the percentage of CRF&NKCC1 double-positive neurons (*P* = 0.0008, *t*_8_ = 5.222, Figure 5J) in the CeA. Collectively, these findings indicate that CUMS increases both NKCC1 protein expression and the percentage of NKCC1-positive CRF-expressing neurons in the CeA.

### NKCC1 contributes to hyperactivity of CeA CRF-expressing neurons by reducing GABAergic inhibition and inducing a depolarizing shift of E_GABA_ in CUMS-induced hypertension.

**Figure 6:**
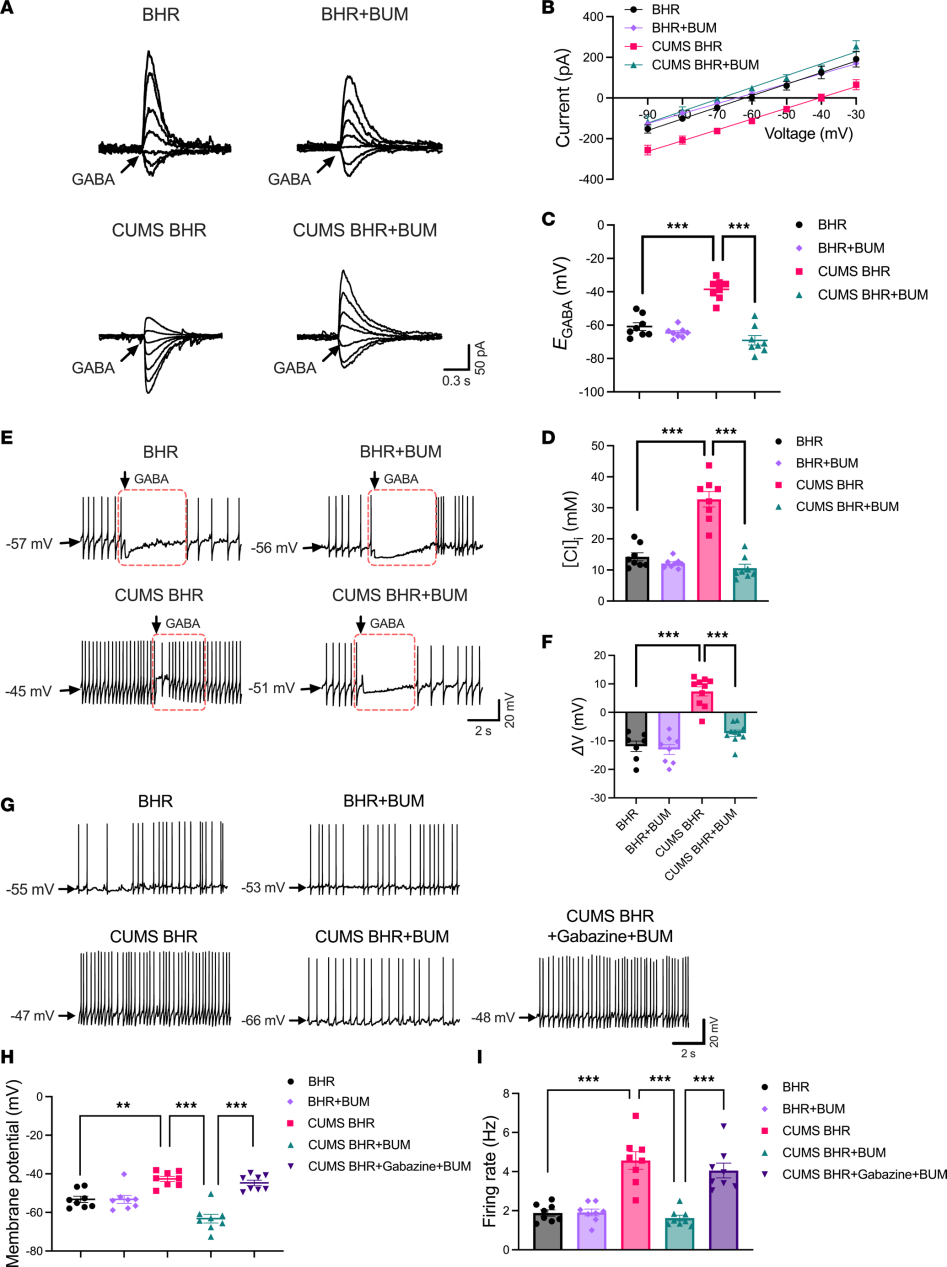
NKCC1 activation contributes to hyperactivity of CeA CRF-expressing neurons by reducing GABAergic inhibition and causing a depolarizing shift of *E*_GABA_ in CUMS BHRs. (**A** and **B**) Original recordings of GABA-induced currents at a series of membrane potentials ranging from –90 to –30 mV (A) and *I-V* plots (**B**) show the effect of bumetanide (BUM, 20 μM) incubation on the GABA-induced currents of CeA CRF-expressing neurons from BHR and CUMS BHR (*n* = 8 neurons in each group). (**C** and **D**) Summary data show the effect of BUM on the *E*_GABA_ (**C**) and derived [Cl^–^]_i_ (**D**) of CRF-expressing neurons in the CeA of BHR and CUMS BHR (*n* = 8 neurons in each group). (**E**) Original recordings show the effect of BUM on changes of membrane potential induced by puff application of GABA (300 μM) to labeled CeA neurons of BHR and CUMS BHR. (**F**) Summary data show the effect of BUM on mean changes of membrane potential (Δ*V*) induced by puff application of GABA to CeA CRF-expressing neurons of BHR and CUMS BHR (*n* = 7 neurons in each group). (**G**) Raw traces show the effect of BUM incubation on spontaneous firing activity of CRF-expressing neurons in the CeA of BHR and CUMS BHR. (**H** and **I**) Summary data show changes in membrane potential (**H**) and firing rate (**I**) of spontaneous firing activity of CRF-expressing neurons in response to BUM incubation in the CeA of BHR and CUMS BHR (*n* = 8 neurons in each group). The arrows show the timepoint of puff application of GABA. Data are expressed as means ± SEM. **P < 0.01, ***P < 0.001. One-way ANOVA followed by Tukey’s post hoc test.

To determine whether upregulated NKCC1 plays a role in the hyperexcitability of CeA CRF-expressing neurons in CUMS BHRs, we incubated the brain slices containing the CeA with bumetanide (BUM, an antagonist of NKCC1, 100 μM, 1 hour) and evaluated the change of *E*_GABA_ of CRF-expressing neurons in CUMS BHRs. BUM pre-treatment significantly decreased the depolarizing shift of *E*_GABA_ (*P* < 0.0001, *F*_3,28_ = 38.32) and restored the [Cl^–^]_i_ (*P* < 0.0001, *F*_3,28_ = 43.45) of CeA CRF-expressing neurons in CUMS BHRs, but it had no effect on the *E*_GABA_ (*P* = 0.6301) and [Cl^–^]_i_ (*P* = 0.7767) in unstressed BHRs (*n* = 8 neurons, Figure 6, A–D). We also assessed the impact of BUM pre-treatment on the GABA responsiveness and spontaneous firing activity of CeA CRF-expressing neurons in both unstressed and CUMS BHRs. BUM incubation hyperpolarized the membrane potential of CeA CRF-expressing neurons following puff application of GABA in CUMS BHRs (*n* = 7 neurons in BHR, *n* = 8 neurons in BHR+BUM, *n* = 10 neurons in CUMS BHR or CUMS BHR+BUM, *P* < 0.0001, *F*_3,31_ = 39.11, Figure 6, E and F). Furthermore, incubation of brain slices with BUM significantly hyperpolarized the membrane potential (*P* < 0.0001, *F*_4,35_ = 21.32) and reduced the firing rate (*P* < 0.0001, *F*_4,35_ = 22.57) of CeA CRF-expressing neurons in CUMS BHRs. Co-incubation with gabazine negated the effects of BUM on firing activity of CeA CRF-expressing neurons (*P* < 0.0001) to the level comparable to those in CUMS BHRs (*n* = 8 neurons, Figure 6, G–I). However, BUM pre-treatment did not affect GABA responsiveness, membrane potential and firing rate of spontaneous firing activity in CeA CRF-expressing neurons of unstressed BHRs (Figure 6, E–I). These results indicate that NKCC1-mediated chloride dysregulation mediates the hyperexcitability of CeA CRF-expressing neurons in CUMS BHRs.

### NKCC1 inhibition reduces heightened sympathetic vasomotor tone in CUMS-induced hypertension.

**Figure 7:**
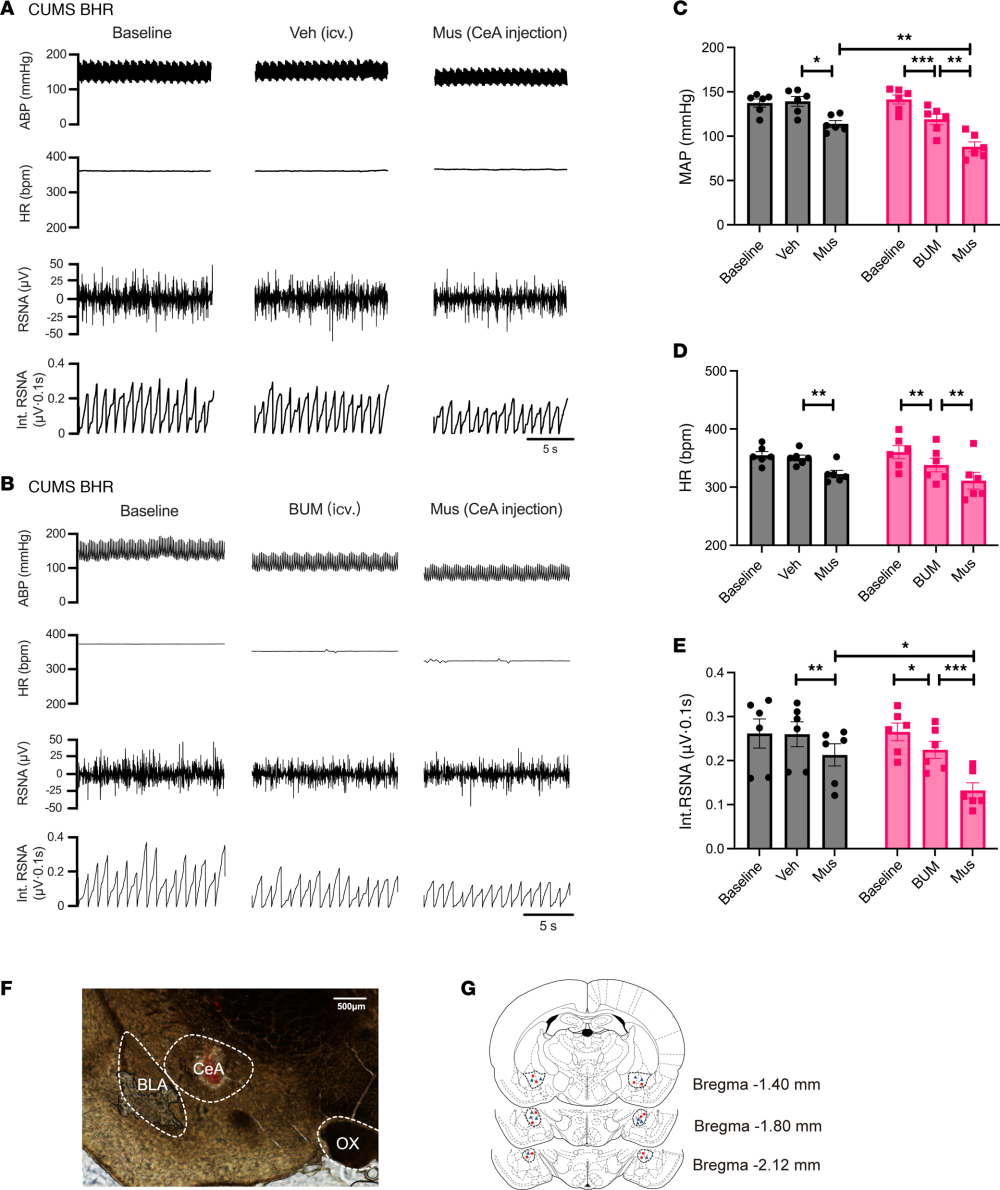
NKCC1 inhibition reduces sympathetic vasomotor tone and enhances sympathoinhibitory responses to GABA_A_ receptor activation in the CeA in chronic stress-induced hypertension. (**A** and **B**) Raw recording traces show the effect of bumetanide (BUM, 200 μmol, 10 μL, icv.) and bilateral injection of muscimol (Mus, 1 nmol, 100 nl, CeA injection) on the ABP, HR, RSNA and Int. RSNA in CUMS BHRs. (**C**–**E**) Summary data show changes of MAP (**C**), HR (**D**) and Int. RSNA (**E**) in response to muscimol microinjection into the CeA following icv. administration of BUM or vehicle (*n* = 6 rats in each group). (**F**) Representative images show the microinjection site in the CeA. (**G**) Schematic drawings show the microinjection sites of muscimol in the CeA pre-treated with vehicle (○) and those microinjection sites pre-treated with BUM (∆) in CUMS BHRs. icv: intracerebroventricular. CeA, central nucleus of amygdala. BLA, basolateral nucleus of amygdala. Veh: vehicle. OX, optic chiasm. Data are expressed as means ± SEM. *P < 0.05, **P < 0.01, ***P < 0.001. One-way ANOVA followed by Tukey’s post hoc test.

We then investigated whether NKCC1 contributes to diminished GABAergic inhibition of the sympathetic outflow in chronic stress-induced hypertension. Intracerebroventricular injection of NKCC1 antagonist BUM (200 μmol, 10 μL) significantly decreased the MAP (*P* = 0.0006, *F*_1.178,5.892_ = 49.54), HR (*P* = 0.0010, *F*_1.061,5.307_ = 37.62) and RSNA (*P* = 0.0254, *F*_1.419,7.096_ = 70.10) in CUMS BHRs. By contrast, intracerebroventricular injection of vehicle had no obvious effect on the MAP (*P* = 0.7238, *F*_1.300,6.501_ = 20.84), HR (*P* = 0.3591, *F*_1.693,8.466_ = 43.91) and RSNA (*P* = 0.9418, *F*_1.162,5.811_ = 18.05) in these animals (*n* = 6 rats, Figure 7, A–E). Microinjection GABA_A_ receptor agonist muscimol (Mus, 1 nmol, 100 nl) into the CeA significantly decreased the MAP (*P* = 0.0116 for vehicle, *P* = 0.0072 for BUM), HR (*P* = 0.0013 for vehicle, *P* = 0.0098 for BUM) and RSNA (*P* = 0.0074 for vehicle, *P* = 0.0003 for BUM) in CUMS BHRs with either vehicle or BUM pre-treatment (*n* = 6 rats, Figure 7, A–E). Notably, muscimol caused a more pronounced decrease in MAP (*P* = 0.0033, *t*_10_ = 3.830) and RSNA (*P* = 0.0254, *t*_10_ = 2.625) in CUMS BHRs with BUM pre-treatment than vehicle pre-treatment (Figure 7, A–E). We also determined the effect of BUM and Mus in unstressed BHRs and CUMS WKY rats. Intra-CeA Mus administration decreased MAP and RSNA in unstressed BHRs, whereas prior BUM injection (icv.) did not affect these parameters (Supplemental Figure 6, A–E). However, neither BUM (icv.) nor intra-CeA Mus affected MAP, HR, or RSNA in CUMS-exposed WKY rats (Supplemental Figure 7, A–E). At the end of each experiment, we confirmed the microinjection locations, and excluded data with microinjection outside the CeA (Figure 7, F and G). These data indicate that elevated NKCC1 activity diminishes GABAergic inhibition of sympathetic outflow in chronic stress-induced hypertension.

## Discussion

Our study shows that CUMS upregulates NKCC1, but not KCC2, in CRF-expressing neurons of the CeA, causing intracellular chloride accumulation and a depolarizing shift of *E*_GABA_ in stress-induced hypertension. This shift converts GABA_A_ receptor responses from inhibitory to excitatory, reducing GABAergic inhibition and increasing the excitability of CeA CRF-expressing neurons, thereby resulting in sympathetic overactivity and sustained hypertension in borderline hypertensive rats.

The interplay between chronic stress and hypertension has long been recognized, with individuals exposed to persistent psychosocial stress being at markedly higher risk for developing hypertension (10, 25, 26). Our findings demonstrate that CUMS produces distinctly different cardiovascular responses in WKY rats versus BHRs. In WKY rats, CUMS induced only a transient increase in MAP that rapidly normalized after stress cessation. In contrast, BHRs exhibited a progressive increase in MAP that stabilized at hypertensive levels during 21-day CUMS, and this hypertension persisted for at least 21 days following stress termination. These results emphasize the critical impact of chronic stress on the development and maintenance of hypertension in borderline hypertension.

While the PVN has traditionally been identified as the primary neural hub linking stress to cardiovascular regulation (11, 12), emerging evidence suggests that extrahypothalamic brain areas, particularly the CeA, also play a critical role (27). Our investigation revealed that CUMS substantially activated CeA CRF-expressing neurons, evidenced by increased delta-FosB immunoreactivity and significantly elevated spontaneous and evoked firing rates. Using optogenetics techniques, we further demonstrated that acute activation of CeA CRF-expressing neurons directly triggered increases in sympathetic nerve activity and blood pressure, whereas chemogenetic inhibition of these neurons prevents stress-induced hypertension. This establishes a causal, not merely correlative, role of CeA CRF-expressing neurons in enhanced sympathetic output and elevated blood pressure. In chronic stress-induced hypertension, PVN CRF-expressing neurons act synergistically with CeA CRF-expressing neurons to enhance stress-driven sympathetic excitation (27). Although PVN CRF-expressing and CeA CRF-expressing neurons are not directly connected, they appear to be functionally linked via the bed nucleus of stria terminalis (BNST), forming a potential CeA-BNST-PVN circuit through which emotional stress may influence sympathetic activity and contribute to the development of hypertension (28).

The CeA is abundantly populated with GABAergic neurons and GABA receptors that modulate responses to stress, addiction, fear, and anxiety (29–32). Previous studies have shown that stress diminishes GABAergic inhibition in basolateral amygdala neurons (33). Our electrophysiological results showed that puff application of GABA depolarized the membrane potential of CeA CRF-expressing neurons in CUMS BHRs. However, in unstressed BHRs, GABA application hyperpolarized CeA CRF-expressing neurons. This opposite membrane potential response indicates a change in the direction of GABA receptor-mediated Cl^–^ flow (GABA-induced current) in CeA CRF-expressing neurons after chronic stress exposure. Chronic stress has been shown to decrease Kv7.2 and Kv7.3 channel expression and reduce M-current in the CeA, thereby increasing CRF-expressing neurons excitability (27). In addition to stress-induced changes in intrinsic excitability through M-current suppression, we identified a mechanism whereby stress disrupts GABA_A_ receptor-mediated synaptic inhibition in CeA CRF-expressing neurons. Thus, impaired Kv7 channel function and NKCC1-mediated GABAergic dysfunction act synergistically (34), rather than independently, to sustain CeA CRF-expressing neurons hyperactivity.

Further investigation of this phenomenon reveals that CUMS significantly depolarized *E*_GABA_ and increased intracellular chloride concentration in CeA CRF-expressing neurons of BHRs. Under these conditions, GABA triggers an outward rather than inward chloride current, effectively converting GABA from an inhibitory to an excitatory neurotransmitter in chronic stress-induced hypertension. Application of gabazine, a selective GABA_A_ receptor antagonist, completely abolished these GABA-induced currents, confirming that GABA_A_ receptors are the primary mediators of this altered chloride conductance. These electrophysiological changes firmly establish that CeA CRF-expressing neuronal hyperexcitability results from compromised GABAergic inhibition and disrupted chloride homeostasis.

The reversed GABA-induced currents are caused by the change of Cl^-^ transporter, mainly NKCC1 and KCC2 in the central nervous system (17). Our biochemical analyses demonstrated that CUMS specifically increased NKCC1 expression in the CeA of BHRs without affecting KCC2 expression. Immunofluorescence staining also revealed a significant increase in the proportion of CRF and NKCC1 double-positive neurons in the CeA following CUMS. Critically, application of bumetanide, a selective NKCC1 inhibitor, normalized intracellular chloride concentration and restored GABAergic inhibition, thereby reducing the excitability of CeA CRF-expressing neurons in CUMS BHRs. These findings identify NKCC1 as a promising therapeutic target for countering stress-induced neurophysiological alterations and potentially preventing or treating hypertension associated with chronic stress.

A limitation of this study is that we did not directly determine whether CeA CRF-expressing neurons are excitatory or inhibitory. However, previous single-cell RNA sequencing data have shown that CRF is predominantly expressed in GABAergic neurons within the CeA (35). Consistently, data from the Allen Brain Cell Atlas also demonstrates co-expression of CRF and GABA in CeA neurons (36). These findings strongly suggest that CeA CRF-expressing neurons are GABAergic. The specific mechanisms by which these GABAergic CRF-expressing neurons modulate downstream circuits to regulate blood pressure and sympathetic activity remains to be elucidated and will be addressed in future studies.

In summary, our study provides compelling evidence that NKCC1-mediated chloride homeostasis dysfunction in CeA CRF-expressing neurons plays a crucial role in heightened sympathetic outflow and hypertension induced by chronic stress. These findings not only advance our understanding of the pathogenesis of stress-related hypertension, but also offer a theoretical foundation and identify potential therapeutic targets for its prevention and treatment.

## Methods

### Sex as a biological variable.

Our study exclusively examined male rats. It is unknown whether the findings are relevant for female rats.

### Animals.

Ten-week-old male normotensive WKY rats and age-matched female SHRs were purchased from Vital River Laboratory Animal Technology Company. BHRs were the first-generation offspring obtained by crossing male WKY rats with female SHRs. We used male BHRs and WKY rats for all subsequent experiments. All animals were housed in the animal facility on a 12-hour light/dark cycle, with free access to food and water (with the exception of the stressors described below).

### Chronic unpredictable mild stress (CUMS).

BHR and WKY rats were subjected to 2 random stressors daily out of 8 possible stressors for 21 days. The stressors include cage rotation, cold isolation at 4°C, light off, light on, forced swim, restraint stress, isolation housing, and food/water deprivation (Supplemental Table 1). The unstressed control BHR and WKY rats were housed in their home cages during the same period.

### Wireless telemeter implantation and blood pressure measurements.

ABP was measured in freely moving, conscious rats using wireless telemeters (Kaha Sciences). Briefly, rats were anesthetized with 2% isoflurane and the catheter connected to the telemetry transmitter was inserted into the abdominal aorta via a right femoral artery incision, then the transmitter was fixed subcutaneously and secured with sutures. The incision was closed in two layers with interrupted sutures. Following surgery, rats received analgesics (meloxicam, 2 mg/kg) and antibiotics (enrofloxacin, 5 mg/kg) for 3 days and were housed individually for a 7-day recovery period. ABP signals were continuously recorded and analyzed by LabChart 8 (AD Instruments) using Powerlab (AD Instruments), and heart rate (HR) values were derived from the ABP pulse signal.

### Immunofluorescence staining.

Rats were deeply anesthetized (isoflurane 5% in O_2_) and transcardially perfused with 200 ml of chilled saline, followed by 4% phosphate-buffered (500 ml, 0.1 mol/L, pH 7.4) paraformaldehyde and 10% sucrose solutions in PBS (200 ml, 0.1 mol/L, pH 7.4). Then the rat was decapitated, and the brain was removed and stored in 4% paraformaldehyde at 4°C for 1 hour, and then immersed in 30% sucrose in PBS at 4°C for at least 2 days. The brain was trimmed and frozen in Tissue-Tek optimal cutting temperature compound. A series of 25 μm coronal sections was obtained with a cryostat microtome (CM1950; Leica Microsystems). The sections were then washed three times with PBS and incubated for 1 hour at 25°C in a blocking solution containing 5% bovine serum albumin and 0.3% Triton X-100 in PBS. Subsequently, the sections were incubated with a mouse anti-delta-FosB antibody (1:50, #sc-398595), a rabbit anti-CRF antibody (1:100, #A1122, Abclonal) or a rabbit anti-NKCC1 antibody (1:100, #13884, Proteintech) overnight at 4°C. After rinsing with PBS again, the sections were incubated with the corresponding Cy 3-conjugated goat anti-mouse antibody (1:500, #115-165-003, Jackson ImmunoResearch), Alexa Fluor 488-conjugated alpaca anti-rabbit antibody (1:500, #611-545-215, Jackson ImmunoResearch) or Cy 3-conjugated goat anti-rabbit antibody (1:500, #111-165-003, Jackson ImmunoResearch) for 2 hours at 25°C. Images were acquired using a confocal microscope (FV3000, Olympus) and processed with the Fluoview software (FV31S, Olympus). Quantification of 3 representative coronal sections per animal was performed using ImageJ software (Version 2.1.0, NIH) by an investigator blinded to group assignment. The “Cell Counter” plugin of ImageJ software was used to count the number of each type of immunofluorescent cells in the CeA.

### Identification of CRF-expressing neurons in the CeA.

We identified CeA CRF-expressing neurons by assessing the specific expression of enhanced green fluorescent protein (eGFP) driven by the rat CRF promoter, as described previously (21). The AAV1/2-CRF-eGFP viral vector (GeneDetect Limited) was bilaterally microinjected into the CeA through a drilled skull hole of rats anesthetized under 2% isoflurane in O_2_. The viral vector was injected using a glass micropipette connected to Nanoject II Microinjectors (Drummond Scientific Company) mounted on a stereotaxic apparatus (RWD Life Science). The tip of the glass micropipette (tip diameter, 20–30 μm) was advanced into the CeA according to the stereotaxic coordinates: 2.3 mm–2.7 mm caudal to the Bregma; 4.3 mm–4.5 mm lateral to the midline; 6.5 mm–6.9 mm ventral to dura. We delivered 2 separate 50 nL injections of the AAV vector into each side of the CeA within 2 minutes. After the injection of viral vectors, the glass pipette was then held in place for an additional 5 minutes before being slowly withdrawn. The rats were returned to their home cages after recovering from anesthesia. The vectors were allowed to be expressed in the CeA for 21–28 days.

### Brain slice preparation and electrophysiological recording.

The rats were rapidly decapitated under 5% isoflurane anesthesia. The brain was quickly removed and sliced in an ice-cold artificial cerebrospinal fluid (aCSF) containing (in mmol/L) 126.0 NaCl, 3.0 KCl, 1.5 MgCl_2_, 2.4 CaCl_2_, 1.2 NaH_2_PO_4_, 11.0 glucose and 26 NaHCO_3_ saturated with 95% O_2_ and 5% CO_2_. Brain slices (300 μm thick) containing the CeA were obtained using a vibrating microtome (VT1000S; Leica Biosystems Inc.). The slices were incubated in the aCSF at 34°C for at least 1 hour before recording.

The eGFP-labeled neurons were identified using epifluorescence illumination and differential interference contrast optics on an upright microscope (BX51 WI, Olympus Optical). The recording electrodes were pulled from borosilicate capillaries (World Precision Instruments) using a micropipette puller (P-97, Sutter Instruments). Electrical signals were amplified with a Multiclamp 700B amplifier (Molecular Devices), filtered at 1–2 kHz, and digitized at 20 kHz by using Digidata 1550 (Molecular Devices).

Performed perforated recordings in current-clamp mode were conducted to measure the spontaneous firing activity of labeled neurons in the CeA. The internal solution consisted of (in mmol/L): 130.0 K-gluconate, 15.0 KCl, 5.0 NaCl, 1.0 MgCl_2_, and 10.0 HEPES (pH adjusted to 7.2 with KOH; 300 mOsmol/L). The tip of the recording pipette was backfilled with the internal pipette solution containing gramicidin (50 μg/ml). Recording of the firing activity of labeled neurons began when the firing activity had reached a steady state for 3–6 minutes. The response of CeA CRF-expressing neurons to GABA was determined by puff application of GABA (300 μM) during recording of firing activity.

The evoked firing activity of labeled neurons was recorded using a current-clamp mode. The firing was evoked by applying stepwise depolarizing currents to the recorded neurons (0 pA to +200 pA, 20 pA increments, 500 ms duration). The recording glass pipette (4–6 MΩ) was filled with an internal solution containing (in mmol/L) 140.0 K-gluconate, 2.0 KCl, 3.0 MgCl_2_, 10.0 HEPES, 5.0 phosphocreatine, 2.0 K-ATP and 0.2 Na-GTP. The pH was adjusted to 7.4 with 1.0 mol/L KOH (290-300 mOsmol/L).

Cl^–^-impermeable gramicidin-perforated recordings were performed using a pipette solution containing (in mM) 140 CsCl, 5 EGTA, and 10 HEPES, pH 7.4 (37, 38). To determine the *E*_GABA_, the GABA currents were evoked by puff application of GABA (300 μM) and recorded at a series of holding potentials ranging from –90 mV to –30 mV at 10 mV increments under voltage-clamp mode. The duration of GABA puff application was less than 15 ms to prevent HCO_3_^–^ currents induced by GABA (39, 40). CGP55845 (2 μM) and tetrodotoxin (TTX, 1 μM) were added to the bath solution to block GABA_B_ receptors and action potential-dependent synaptic activity, respectively.

GABA was purchased from Sigma-Aldrich. Bumetanide, gabazine and TTX were obtained from Cayman Chemical.

### Optogenetic viral vectors injection and fiber-optic implantation.

We employed a dual viral vector strategy to selectively express ChR2 in CeA CRF-expressing neurons. The first AAV vector carried a double-inverted open (DIO) reading frame and modified ChR2 fused to eYFP, driven by human Synapsin I (hSyn) promoter (AAV2-hSyn-ChR2-DIO-eYFP). The second AAV vector, which expressed Cre recombinase specifically in CRF-expressing neurons, utilized a full-length rat CRF promoter to drive the expression of an optimized Cre recombinase gene (AAV2-CRF-Cre, Genedetect). A 100 nl mixture of the 2 viral vectors was bilaterally microinjected into the CeA using a glass micropipette following a previously described procedure (41). The glass micropipette was slowly advanced to 6.8 mm ventral to the dura, then retracted to 6.6 mm ventral to the dura to facilitate viral diffusion and minimize backflow along the injection track. Optical fibers (200 μm O.D., 0.22 NA; Inper Ltd) were then implanted bilaterally into the CeA (about 6.5 mm ventral to the dura, 100–300 μm above the viral injection site), and secured to the skull with bone screws and dental cement. A period of 3 to 4 weeks was allowed for ChR2 to be expressed in the CeA CRF-expressing neurons. A fiber-optic rotary joint was used to allow simultaneous photostimulation of both CeA regions, as previously described (42). Blue light photostimulation (470 nm, 5 mW, duration 7 seconds and delay 3 seconds in 10 seconds, total 60 seconds) at 10 Hz, 20 Hz, or 40 Hz using an intelligent optogenetic system (Aurora-300, NEWDOON) to selectively activate CeA CRF-expressing neurons.

### Chemogenetic viral vectors injection and CNO treatment.

To specifically inhibit CeA CRF-expressing neurons, a chemogenetic approach utilizing Designer Receptors Exclusively Activated by Designer Drugs (DREADDs) was employed. AAV2-CRF-Cre and AAV (PHP.eB)-hsyn-DIO-hM4Di-mCherry (Genechem) mixture (100 nl) was bilaterally microinjected into the CeA. Following 3 weeks for viral expression, compound Clozapine N-oxide (CNO) was administered intraperitoneally (i.p.) to inhibit CeA CRF-expressing neurons during the CUMS period. Control animals received only AAV (PHP.eB)-hsyn-DIO-hM4Di-mCherry injection and the same CNO treatment.

### Recording of ABP and RSNA.

Rats were anesthetized by intraperitoneal injection of α-chloralose (60 mg/kg) and urethane (800 mg/kg). The ABP was continuously monitored via a catheter inserted in the right femoral artery, and HR was derived from the pulsatile pressure wave. A branch of the left renal postganglionic sympathetic nerve was isolated under a microscope and cut distally. At the end of each experiment, the proximal end of the nerve was crushed to eliminate the nerve discharge, and the residual noise level was measured. RSNA was rectified and integrated offline after background noise subtraction. Integrated RSNA (Int. RSNA) was calculated from raw RSNA using Spike2 software. Nerve signals were amplified and bandpass filtered using an AC amplifier (model NL125/NL126; NeuroLog System). RSNA and ABP were recorded using a 14-bit analog-to-digital converter and analyzed with Spike2 software (Cambridge Electronic Design). The MAP was calculated from the recorded systolic and diastolic blood pressures.

To assess the impact of activating CeA CRF-expressing neurons on ABP, HR and RSNA, optogenetic stimulation was applied to the ChR2-expressing CeA CRF neurons of unstressed BHRs.

For intracerebroventricular or CeA microinjection, the rats were fixed on a stereotaxic apparatus. The coordinates used for intracerebroventricular injection were 1.0 mm caudal to the bregma, 1.5 mm lateral to the midline and 3.0 mm ventral to the dura. For CeA microinjection, the coordinates were 2.3 mm–2.7 mm caudal to the bregma, 4.2 mm–4.3 mm lateral to the midline and 6.5 mm–6.9 mm ventral to the dura. After the brain was exposed, a microinjection pipette was advanced into the lateral ventricle or CeA according to the stereotaxic coordinates as above. Bumetanide (200 μmol, 10 μL) was administered intracerebroventricularly, and muscimol (1 nmol, 100 nl) was bilaterally microinjected into the CeA to observe the changes in MAP and RSNA. A 5% DiI fluorescent tracer was added to the microinjection solution to verify injection sites post-experiment. Only animals with injections entirely confined to the CeA were included in the analysis, rats with misplaced microinjections outside of the CeA were excluded from data analysis (*n* = 2 rats in CUMS BHR, *n* = 1 rat in BHR and CUMS WKY).

### Western immunoblotting.

Membrane proteins were extracted from the CeA, FC, and RVLM tissues using membrane protein extraction kits (PK10015, Proteintech) in the presence of a protease inhibitor cocktail (PR20032, Proteintech) following the manufacturer’s instructions. The samples were subjected to 10% Tris–HCl SDS-PAGE and then transferred to a polyvinylidene difluoride membrane (Millipore, Sigma). The membranes were incubated with a rabbit anti-NKCC1 antibody (1:1,000, #13884, Proteintech), a rabbit anti-KCC2 antibody (1:1,000, #19565, Proteintech), rabbit anti-GABA_A_ receptor antibody (1:2,000, #12410, Proteintech) or rabbit anti-Na-K-ATPase antibody (1:5,000, #14418, Proteintech) overnight at 4°C. After washing with TBST, the membranes were incubated with a goat anti-rabbit IgG/HRP secondary antibody (1:5,000, #SA00001, Proteintech) or a goat anti-mouse IgG/HRP secondary antibody (1:5,000, #SA00001, Proteintech) for 1 hour at 25°C. Protein bands were visualized using an ECL kit (#RP-WA0601, Report Bio&Technology Co., Ltd), and the images were captured with a ChemiScope S6 (CLINX). Target protein bands were normalized to the Na-K-ATPase bands on the same blot.

### Corticosterone (CORT) level measurement.

Serum CORT level was quantified using a commercially available enzyme-linked immunosorbent assay (ELISA) kit (#MM-0574R1, Meimian). Briefly, blood samples were centrifuged, and serum was collected and stored at –80°C until analysis. All serum samples and kit reagents were brought to room temperature prior to the assay. Samples were appropriately diluted according to the manufacturer’s instructions and added to antibody-coated microplate wells. After incubation and washing, a biotin-conjugated detection antibody was added, followed by a streptavidin-HRP conjugate. The reaction was developed with TMB substrate, stopped with stop solution, and the optical density was immediately measured at 450 nm using a microplate reader. CORT concentration was calculated by interpolating from a standard curve generated with known CORT standards. All samples were assayed in duplicate.

### GABA level measurement.

GABA levels in CeA tissue were quantified using liquid chromatography-tandem mass spectrometry (LC-MS/MS, AB SCIEX Strap 6500 plus). Briefly, rats CeA tissue samples were homogenized in a chilled acetonitrile solution. After protein precipitation and centrifugation, the supernatants were collected and derivatized to enhance chromatographic separation and detection sensitivity. The derivatives were separated on a reversed-phase C18 column using a gradient elution and analyzed by MS/MS in positive electrospray ionization and multiple reaction monitoring (MRM) mode. GABA concentrations were determined by comparing the peak area ratio of analyte to internal standard against a linear calibration curve and normalized to total protein content.

### Statistics.

Data are presented as means ± SEM. Data collection was randomized, and electrophysiological data and immunofluorescence images were analyzed by investigators who were blinded from treatment groups. For electrophysiological recordings, only 1 neuron in each brain slice was recorded, and at least 3 rats were used for each group. The membrane potential and firing rate of neurons were analyzed using Clampfit 11 software. The *E*_GABA_ was determined by using linear regression to calculate a best-fit line for the voltage dependence of GABA-induced currents. The intercept of the current-voltage line with the abscissa was taken as the *E*_GABA_, and then [Cl^–^]_i_ was calculated using the Nernst equation. The MAP, RSNA and HR were analyzed using Spike2 software (Cambridge Electronic Design). Two-tailed Student’s *t*-test was used for comparisons between 2 groups. One-way ANOVA followed by Tukey’s post hoc test was used for comparisons among more than 2 groups. Repeated Measures of 2-way ANOVA followed by Tukey’s post hoc multiple comparison tests were performed to compare daily MAP and HR. Statistical analyses were performed using Prism 9 software (GraphPad Software Inc.). *P* < 0.05 was considered statistically significant.

### Study approval.

All experiments were carried out in compliance with the US National Institutes of Health guidelines for the Care and Use of Laboratory Animals, and all procedures were reviewed and approved by the Ethics Committee for the Use of Experimental Animals at Hebei Medical University (IACUC-Hebmu-2024030).

### Data availability.

Values for all data points in graphs are reported in the Supporting Data Values file. Data are available upon request.

## Author contributions

HYM, YZ, XG, and QZ perform the experiments and acquired and analyzed the data. YL, PY, and YG acquired and analyzed the data. HYM interpreted the data and drafted the manuscript. HJM and HYM conceived the experiment. HJM and YW revised the manuscript and supervised the work.

## Funding support

This study was supported by the following sources.

- National Natural Science Foundation of China (31971044, 82571813).
- Natural Science Foundation of Hebei Province (H2019206325, C2021106015).
- Projects for the introduction of overseas students in Hebei Province (C20220507).
- Key laboratory of Neural and Vascular Biology, Ministry of Education of China (NV20210004, NV20230019).
- Natural Science Foundation of Hebei Province for Innovative Research Group Project (H2025206897).
- Hebei Postgraduate Innovation Grant Program (CXZZBS2025103, XCXZZB202317, CXZZBS2025101).
- The Open Fund of Key Laboratory of Medical Electrophysiology, Ministry of Education, Southwest Medical University (No. KeyME-2024-02).

## Supplementary Material

Supplemental data

Unedited blot and gel images

Supporting data values

## Version 1 03/16/2026

Electronic publication

## References

[1] Carey RM (2022). Treatment of hypertension: a review. JAMA.

[2] Rapsomaniki E (2014). Blood pressure and incidence of twelve cardiovascular diseases: lifetime risks, healthy life-years lost, and age-specific associations in 1·25 million people. Lancet.

[3] Parati G (2015). Antihypertensive therapy in 2014: Linking pathophysiology to antihypertensive treatment. Nat Rev Cardiol.

[4] Spruill TM (2010). Chronic psychosocial stress and hypertension. Curr Hypertens Rep.

[5] Fontes MAP (2023). Neurogenic background for emotional stress-associated hypertension. Curr Hypertens Rep.

[6] Hoffmann TJ (2017). Genome-wide association analyses using electronic health records identify new loci influencing blood pressure variation. Nat Genet.

[7] Hunt RA, Tucker DC (1993). Developmental sensitivity to high dietary sodium chloride in borderline hypertensive rats. Hypertension.

[8] Lawler JE (1988). The borderline hypertensive rat: a model for studying the mechanisms of environmentally induced hypertension. Health Psychol.

[9] Sanders BJ, Lawler JE (1992). The borderline hypertensive rat (BHR) as a model for environmentally-induced hypertension: a review and update. Neurosci Biobehav Rev.

[10] Zhou JJ (2021). α2δ-1-Dependent NMDA receptor activity in the hypothalamus is an effector of genetic-environment interactions that drive persistent hypertension. J Neurosci.

[11] Elsaafien K (2021). Identification of novel cross-talk between the neuroendocrine and autonomic stress axes controlling blood pressure. J Neurosci.

[12] Deussing JM, Chen A (2018). The corticotropin-releasing factor family: physiology of the stress response. Physiol Rev.

[13] Wittmann G (2005). Glutamatergic innervation of corticotropin-releasing hormone- and thyrotropin-releasing hormone-synthesizing neurons in the hypothalamic paraventricular nucleus of the rat. Brain Res.

[14] Kakizawa K (2016). A novel GABA-mediated corticotropin-releasing hormone secretory mechanism in the median eminence. Sci Adv.

[15] Ben-Ari Y (2007). GABA: a pioneer transmitter that excites immature neurons and generates primitive oscillations. Physiol Rev.

[16] Liu ZP (2014). Chronic stress impairs GABAergic control of amygdala through suppressing the tonic GABAA receptor currents. Mol Brain.

[17] Blaesse P (2009). Cation-chloride cotransporters and neuronal function. Neuron.

[18] Pressey JC (2023). Chloride transporters controlling neuronal excitability. Physiol Rev.

[19] Lam P (2023). Cation-chloride cotransporters KCC2 and NKCC1 as therapeutic targets in neurological and neuropsychiatric disorders. Molecules.

[20] Orlov SN (2015). NKCC1 and NKCC2: the pathogenetic role of cation-chloride cotransporters in hypertension. Genes Dis.

[21] Gao Y (2017). Chronic unpredictable mild stress induces loss of GABA inhibition in corticotrophin-releasing hormone-expressing neurons through NKCC1 upregulation. Neuroendocrinology.

[22] Charmandari E (2005). Endocrinology of the stress response. Annu Rev Physiol.

[23] Ye ZY (2012). NKCC1 upregulation disrupts chloride homeostasis in the hypothalamus and increases neuronal activity-sympathetic drive in hypertension. J Neurosci.

[24] Schulte JT (2018). Chloride transporters and GABA polarity in developmental, neurological and psychiatric conditions. Neurosci Biobehav Rev.

[25] Hatton DC (1993). Stress-induced hypertension in the borderline hypertensive rat: stimulus duration. Physiol Behav.

[26] Esler M (2008). Chronic mental stress is a cause of essential hypertension: presence of biological markers of stress. Clin Exp Pharmacol Physiol.

[27] Sheng ZF (2023). Corticotropin-releasing hormone neurons in the central nucleus of amygdala are required for chronic stress-induced hypertension. Cardiovasc Res.

[28] Kubota N (2023). Neural pathways from the central nucleus of the amygdala to the paraventricular nucleus of the hypothalamus are involved in induction of yawning behavior due to emotional stress in rats. Behav Brain Res.

[29] Domi E (2023). Activation of GABA_B_ receptors in central amygdala attenuates activity of PKCδ + neurons and suppresses punishment-resistant alcohol self-administration in rats. Neuropsychopharmacology.

[30] Pomrenze MB (2019). Dissecting the roles of GABA and neuropeptides from rat central amygdala CRF neurons in anxiety and fear learning. Cell Rep.

[31] Varodayan FP (2020). PACAP regulation of central amygdala GABAergic synapses is altered by restraint stress. Neuropharmacology.

[32] Partridge JG (2016). Stress increases GABAergic neurotransmission in CRF neurons of the central amygdala and bed nucleus stria terminalis. Neuropharmacology.

[33] Rodriguez Manzanares PA (2005). Previous stress facilitates fear memory, attenuates GABAergic inhibition, and increases synaptic plasticity in the rat basolateral amygdala. J Neurosci.

[34] Tsentsevitsky AN (2024). GABA receptors and K_v_7 channels as targets for GABAergic regulation of acetylcholine release in frog neuromuscular junction. Neurochem Res.

[35] Hochgerner H (2023). Neuronal types in the mouse amygdala and their transcriptional response to fear conditioning. Nat Neurosci.

[36] Lein ES (2007). Genome-wide atlas of gene expression in the adult mouse brain. Nature.

[37] Ebihara S (1995). Gramicidin-perforated patch recording: GABA response in mammalian neurones with intact intracellular chloride. J Physiol.

[38] Kyrozis A, Reichling DB (1995). Perforated-patch recording with gramicidin avoids artifactual changes in intracellular chloride concentration. J Neurosci Methods.

[39] Grover LM (1993). Role of HCO3- ions in depolarizing GABAA receptor-mediated responses in pyramidal cells of rat hippocampus. J Neurophysiol.

[40] Kaila K (1997). Long-lasting GABA-mediated depolarization evoked by high-frequency stimulation in pyramidal neurons of rat hippocampal slice is attributable to a network-driven, bicarbonate-dependent K+ transient. J Neurosci.

[41] Soriano JE (2023). Longitudinal interrogation of sympathetic neural circuits and hemodynamics in preclinical models. Nat Protoc.

[42] Dedic N (2018). Chronic CRH depletion from GABAergic, long-range projection neurons in the extended amygdala reduces dopamine release and increases anxiety. Nat Neurosci.

