# Supplementary material for: Chloride homeostasis dysfunction drives hyperactivation of corticotropin-releasing factor-expressing neurons in the amygdala in stress-induced hypertension
Source: J Clin Invest. 2026 Mar 16;136(6):e195536. doi: 10.1172/JCI195536 (PMC12987618; doi:10.1172/JCI195536)
Supplement: Supplemental data [file jci-136-195536-s008.pdf]

Chloride homeostasis dysfunction drives hyperactivation of corticotropin-releasing factor-expressing neurons in the amygdala in stress-induced hypertension

Hongyu Ma[1,2], Ying Zhang[1], Xinqi Guo[1], Qiyue Zhao[1], Peiyun Yang[1], Yan Liu[3], Yue Guan[1], Yan Wei[4], Huijie Ma[1,2,5]

[1]Department of Physiology, Hebei Medical University, Shijiazhuang 050017, Hebei, China

[2]The Key Laboratory of Neural and Vascular Biology, Ministry of Education, Hebei Medical University, Shijiazhuang 050017, China

[3]Departments of Endocrinology, The Third Hospital of Hebei Medical university, Shijiazhuang, Hebei, 050017, China

[4]Key Laboratory of Medical Electrophysiology, Ministry of Education & Medical Electrophysiological Key Laboratory of Sichuan Province, Institute of Cardiovascular Research, Southwest Medical University, Luzhou 646000, China

[5]Hebei Key Laboratory of Brain Science and Brain-Inspired Intelligence, Shijiazhuang 050017, Hebei, China

Correspondence to: Huijie Ma, MD, PhD, Department of Physiology, Hebei Medical University, Shijiazhuang 050017, Hebei, China. Email: huijiema@hebmu.edu.cn; Yan Wei, Key Laboratory of Medical Electrophysiology, Ministry of Education & Medical Electrophysiological Key Laboratory of Sichuan Province, Institute of Cardiovascular Research, Southwest Medical University, Luzhou 646000, China. Email: weiyan.1111@swmu.edu.cn

Supplementary figures and legends

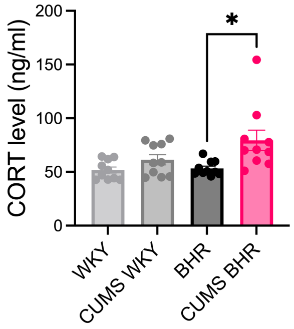

Supplementary figure 1. Corticosterone (CORT) levels in the serum of WKY, CUMS WKY, BHR and CUMS BHR.

n = 10 rats per group. $^{*}P < 0.05$, One-way ANOVA followed by Tukey's *post hoc* test.

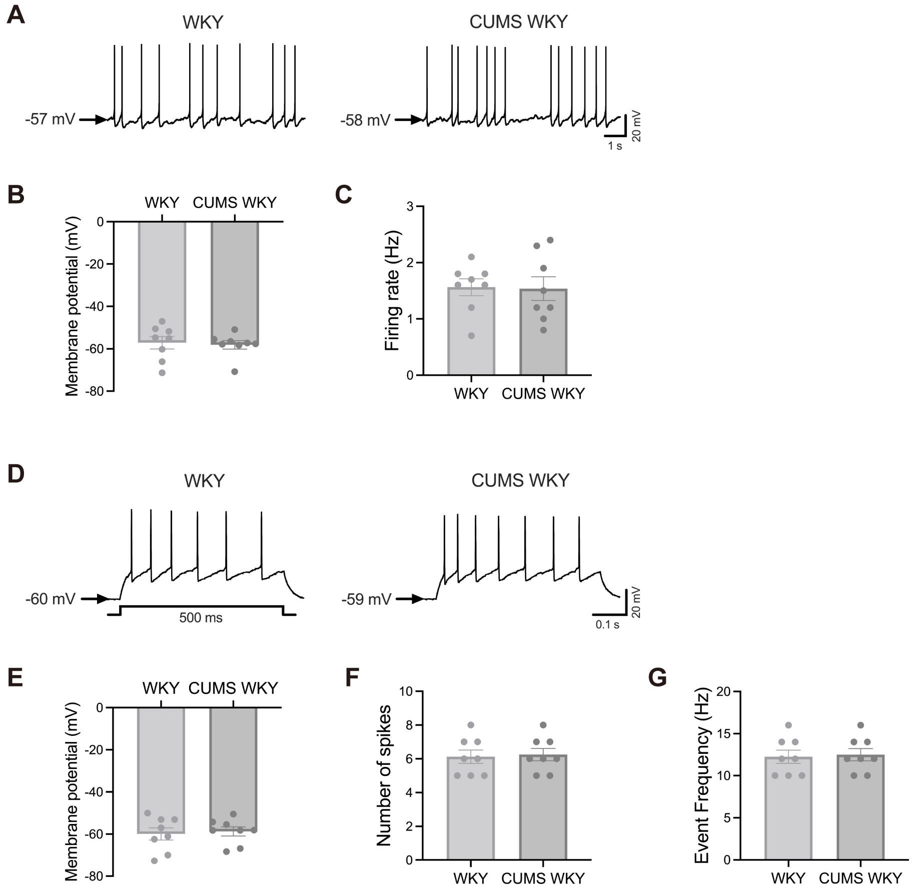

Supplementary figure 2. Firing activity of CeA CRF-expressing neurons in WKY and CUMS WKY.

A. Original traces of spontaneous firing activity of CRF-expressing neurons in the CeA of WKY and CUMS WKY. **B-C.** Membrane potential (B) and firing rate (C) of spontaneous firing activity (*n* = 8 neurons per group). **D.** Original traces of currents evoked firing activity of CRF-expressing neurons in the CeA of WKY and CUMS WKY. **E-G.** Membrane potential (E), number of spikes (F), and event frequency (G) of evoked firing activity (*n* = 8 neurons per group). Two-tailed Student's *t*-test.

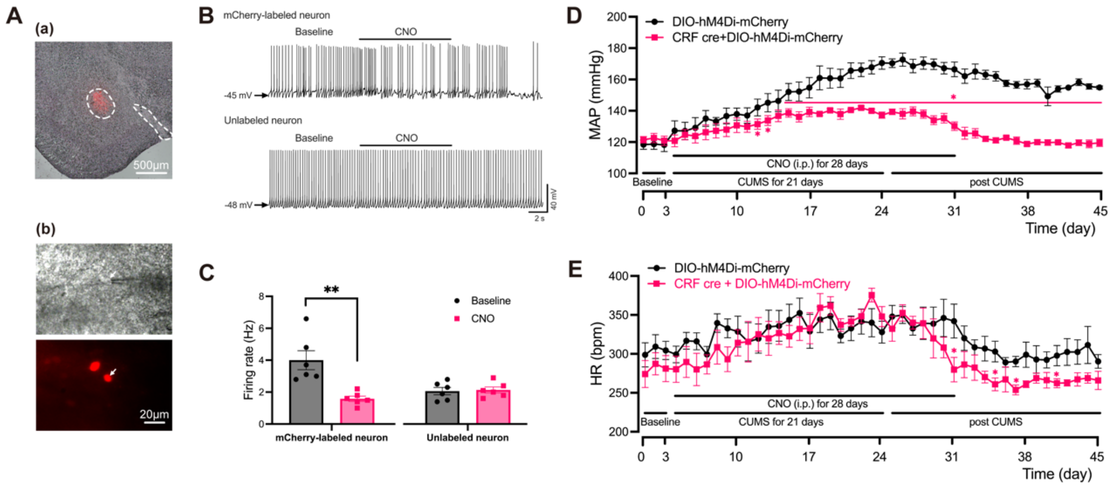

Supplementary figure 3. Chemogenetic inhibition of CeA CRF-expressing neurons improved chronic stress induced hypertension.

A. Representative images show hM4Di-mCherry expression within the CeA (a) and the mCherry-labeled neuron (white arrow) with an attached recording electrode (b). **B-C.** Original traces (B) and summary data (C) show the effects of CNO bath application (10 μM) on the spontaneous firing activity of both mCherry-labeled and unlabeled neurons in the CeA of CUMS BHR (*n* = 6 neurons per group). $^{**}P < 0.01$, Two-tailed paired Student's *t*-test. **D-E.** Summary data show the effects of CNO administration (1 mg/kg, i.p.) on MAP (D) and HR (E) of BHRs before, during and after CUMS (*n* = 6 rats per group). $^{*}P < 0.05$ versus respective values in DIO-hM4Di-mCherry group. Repeated measures of two-way ANOVA with Tukey's multiple comparison tests.

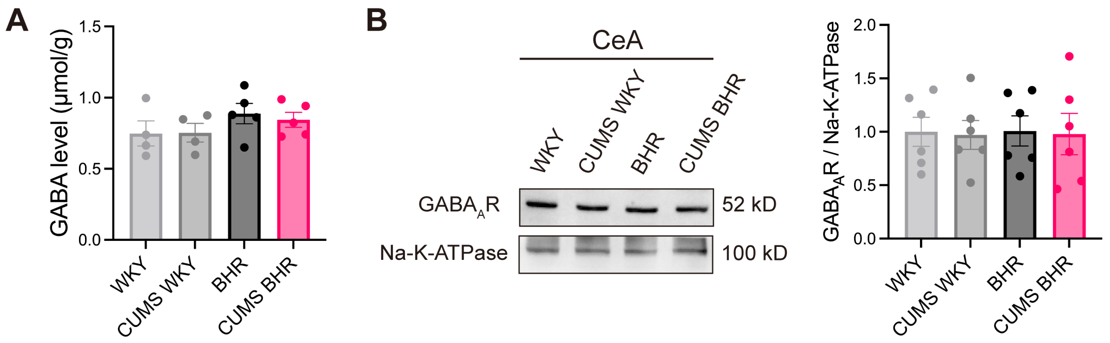

Supplementary figure 4. GABA and GABA$_A$ receptor (GABA$_A$R) levels in the CeA of WKY, CUMS WKY, BHR and CUMS BHR.

A. GABA levels in the CeA of WKY, CUMS WKY, BHR and CUMS BHR (n = 4 samples from 8 rats in WKY rats and CUMS WKY, n = 5 samples from 10 rats in BHR and CUMS BHR). **B.** Representative immunoblots and quantification of GABA$_A$ receptor protein expression in the CeA of WKY, CUMS WKY, BHR and CUMS BHR (n = 6 rats in each group). One-way ANOVA followed by Tukey's *post hoc* test.

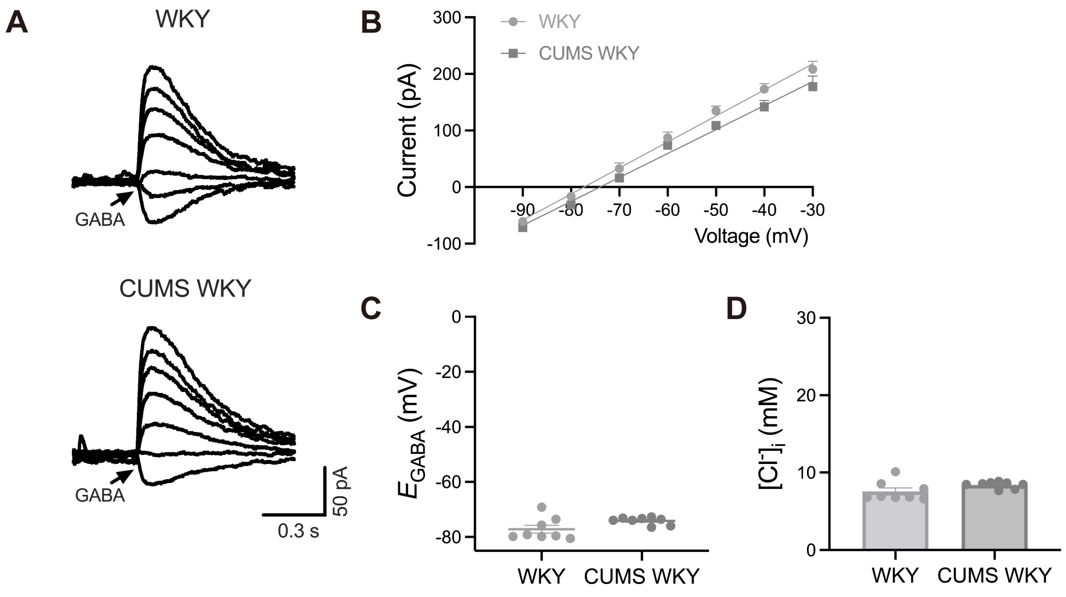

Supplementary figure 5. GABA reversal potential and intracellular chloride concentration of CeA CRF-expressing neurons in WKY and CUMS WKY.

A-B. Representative perforated patch recordings of GABA-induced currents at a series of membrane potentials ranging from -90 to -30 mV (A) and *I-V* plots (B) show the E_{GABA} of CeA CRF-expressing neurons from WKY and CUMS WKY. **C-D.** Summary data show changes in E_{GABA} (C) and derived [Cl$^-$]$_i$ (D) in CeA CRF-expressing neurons (n = 8 neurons in each group). Two-tailed Student's t-test. The arrows show the timepoint of puff application of GABA.

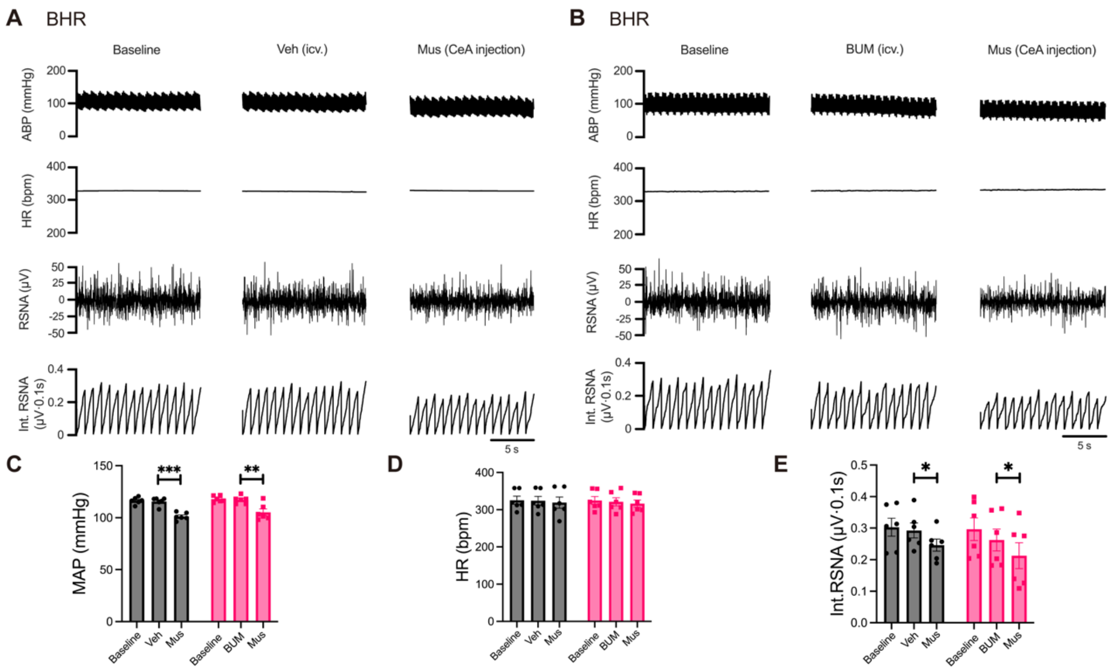

Supplementary figure 6. Effect of NKCC1 antagonist bumetanide on $GABA_A$ receptor response and changes in ABP and sympathetic activity in BHRs.

A & B. Raw recording traces show the effect of bumetanide (BUM, 200 μmol, 10 μL, icv.) and bilateral injection of muscimol (Mus, 1 nmol, 100 nl, CeA injection) on the ABP, HR, RSNA and Int. RSNA in BHRs. **C-E.** Summary data show changes of MAP (C), HR (D) and Int. RSNA (E) in response to muscimol microinjection into the CeA following icv. administration of BUM or vehicle (*n* = 6 rats in each group). $^{*}P < 0.05$, $^{**}P < 0.01$, $^{***}P < 0.001$. One-way ANOVA followed by Tukey's *post hoc* test.

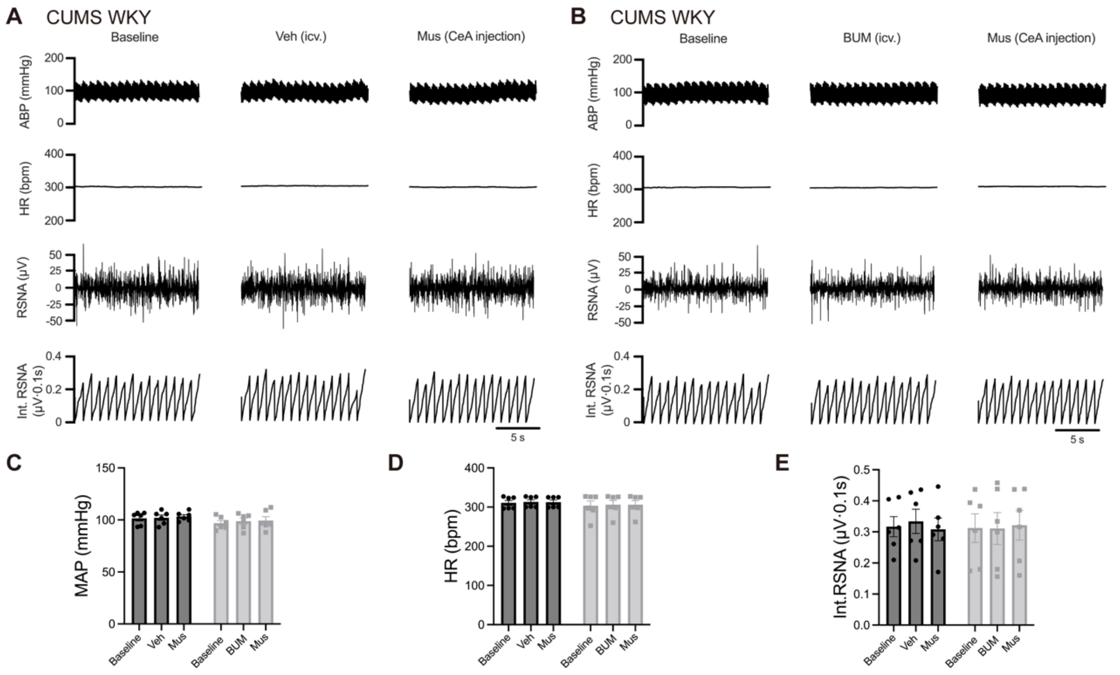

Supplementary figure 7. Effect of NKCC1 antagonist bumetanide on $GABA_A$ receptor response and changes in ABP and sympathetic activity in CUMS WKY.

A & B. Raw recording traces show the effect of bumetanide (BUM, 200 μmol, 10 μL, icv.) and bilateral injection of muscimol (Mus, 1 nmol, 100 nl, CeA injection) on the ABP, HR, RSNA and Int. RSNA in CUMS WKY. **C-E.** Summary data show changes of MAP (C), HR (D) and Int. RSNA (E) in response to muscimol microinjection into the CeA following icv. administration of BUM or vehicle (n = 6 rats in each group). One-way ANOVA followed by Tukey's *post hoc* test.

Supplementary table

Supplementary table 1. List of CUMS procedure details

Day	Time	Stressor	Duration	Time	Stressor	Duration
1	9:00 A.M.	Swim	3 min	10:00 A.M.	Cold	60 min
2	10:00 A.M.	Rotation	60 min	7:00 P.M.	Food deprivation	12 h
3	5:00 P.M.	Rotation	60 min	7:00 P.M.	Isolation	12 h
4	9:00 A.M.	Swim	3 min	7:00 P.M.	Light on	12 h
5	10:00 A.M.	Cold	60 min	7:00 P.M.	Light on	12 h
6	10:00 A.M.	Cold	60 min	3:00 P.M.	Restrain	2 h
7	9:00 A.M.	Swim	3 min	8:00 A.M.	Light off	12 h
8	7:00 P.M.	Isolation	12 h	7:00 P.M.	Food deprivation	12 h
9	2:00 P.M.	Restrain	2 h	7:00 P.M.	Food deprivation	12 h
10	10:00 A.M.	Swim	3 min	11:00 A.M.	Rotation	60 min
11	3:00 P.M.	Cold	60 min	7:00 P.M.	Light on	12 h
12	9:00 A.M.	Restrain	2 h	8:00 A.M.	Light off	12 h
13	9:00 A.M.	Swim	3 min	3:00 P.M.	Restrain	2 h
14	10:00 A.M.	Rotation	60 min	11:00 A.M.	Cold	60 min
15	9:00 A.M.	Cold	60 min	7:00 P.M.	Light on	12 h
16	2:00 P.M.	Rotation	60 min	4:00 P.M.	Restrain	2 h
17	2:00 P.M.	Cold	60 min	7:00 P.M.	Light on	12 h
18	8:00 P.M.	Isolation	12 h	8:00 P.M.	Food deprivation	12 h
19	10:00 A.M.	Rotation	60 min	3:00 P.M.	Restrain	2 h
20	3:00 P.M.	Restrain	2 h	7:00 P.M.	Light on	12 h
21	9:00 A.M.	Cold	60 min	7:00 A.M.	Light off	12 h
