# Supplementary material for: Chloride homeostasis dysfunction drives hyperactivation of corticotropin-releasing factor-expressing neurons in the amygdala in stress-induced hypertension
Source: J Clin Invest. 2026 Mar 16;136(6):e195536. doi: 10.1172/JCI195536 (PMC12987618; doi:10.1172/JCI195536)

- Full unedited gels for Figure 5A

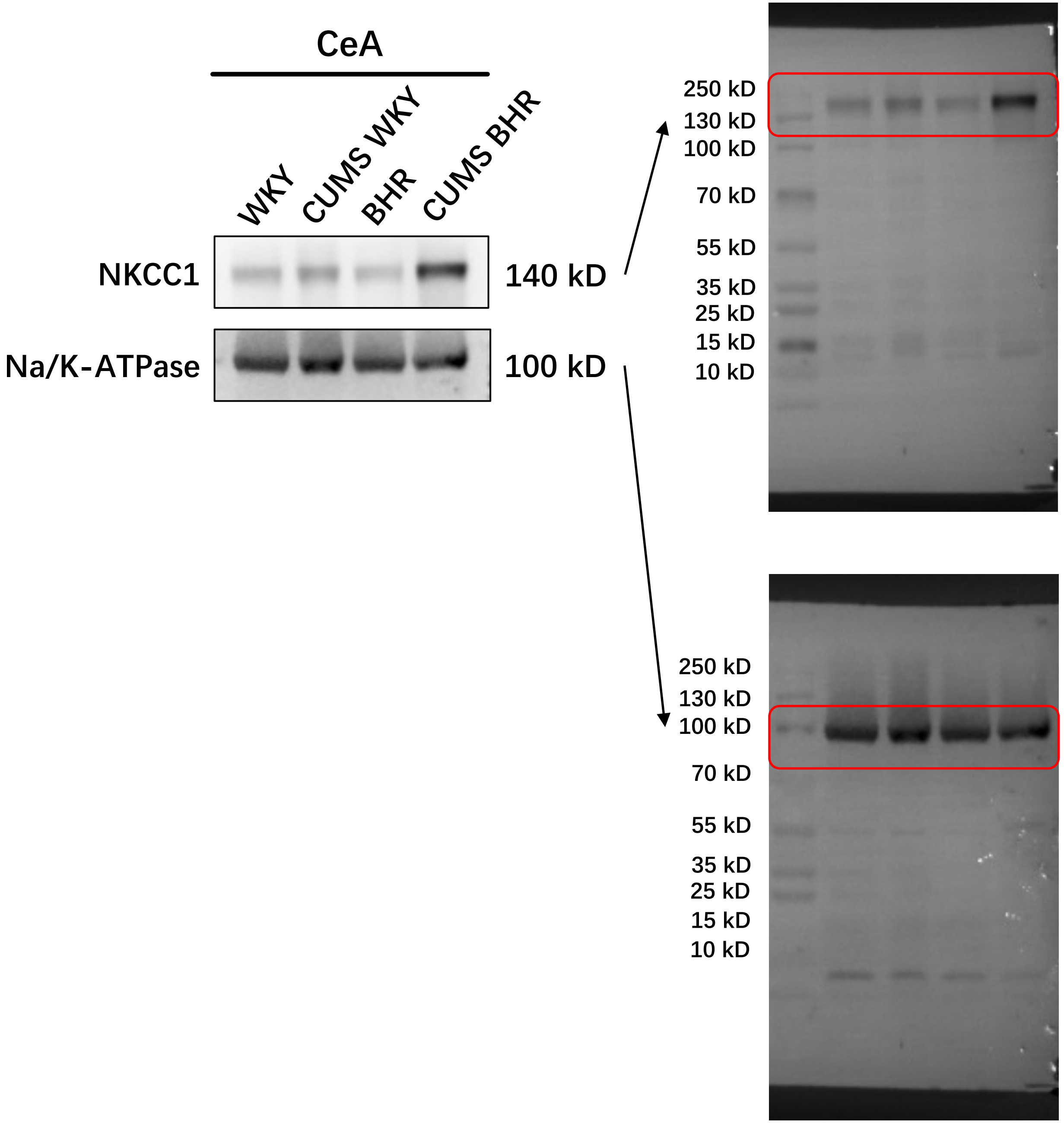

- Full unedited gels for Figure 5B

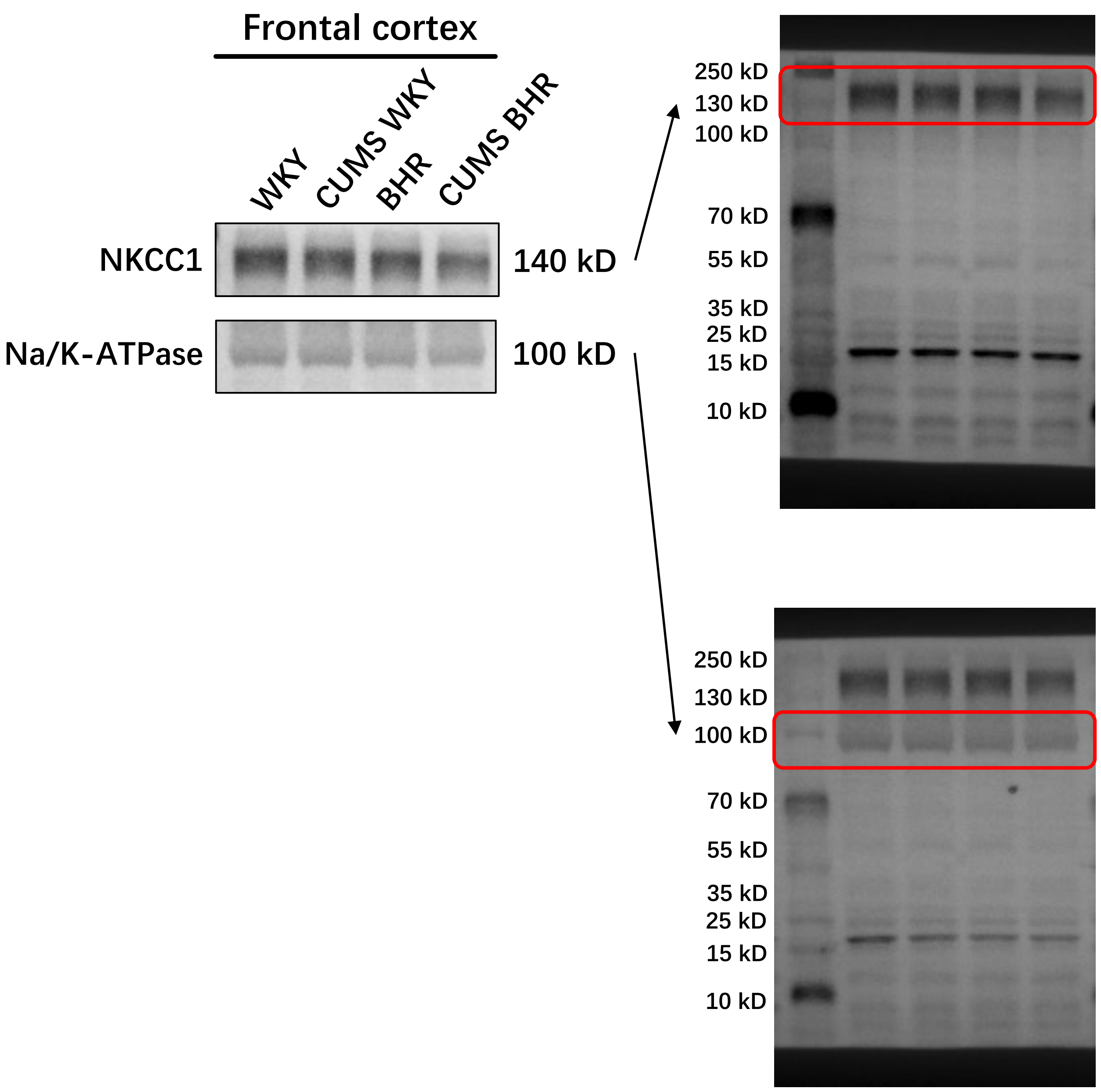

- Full unedited gels for Figure 5C

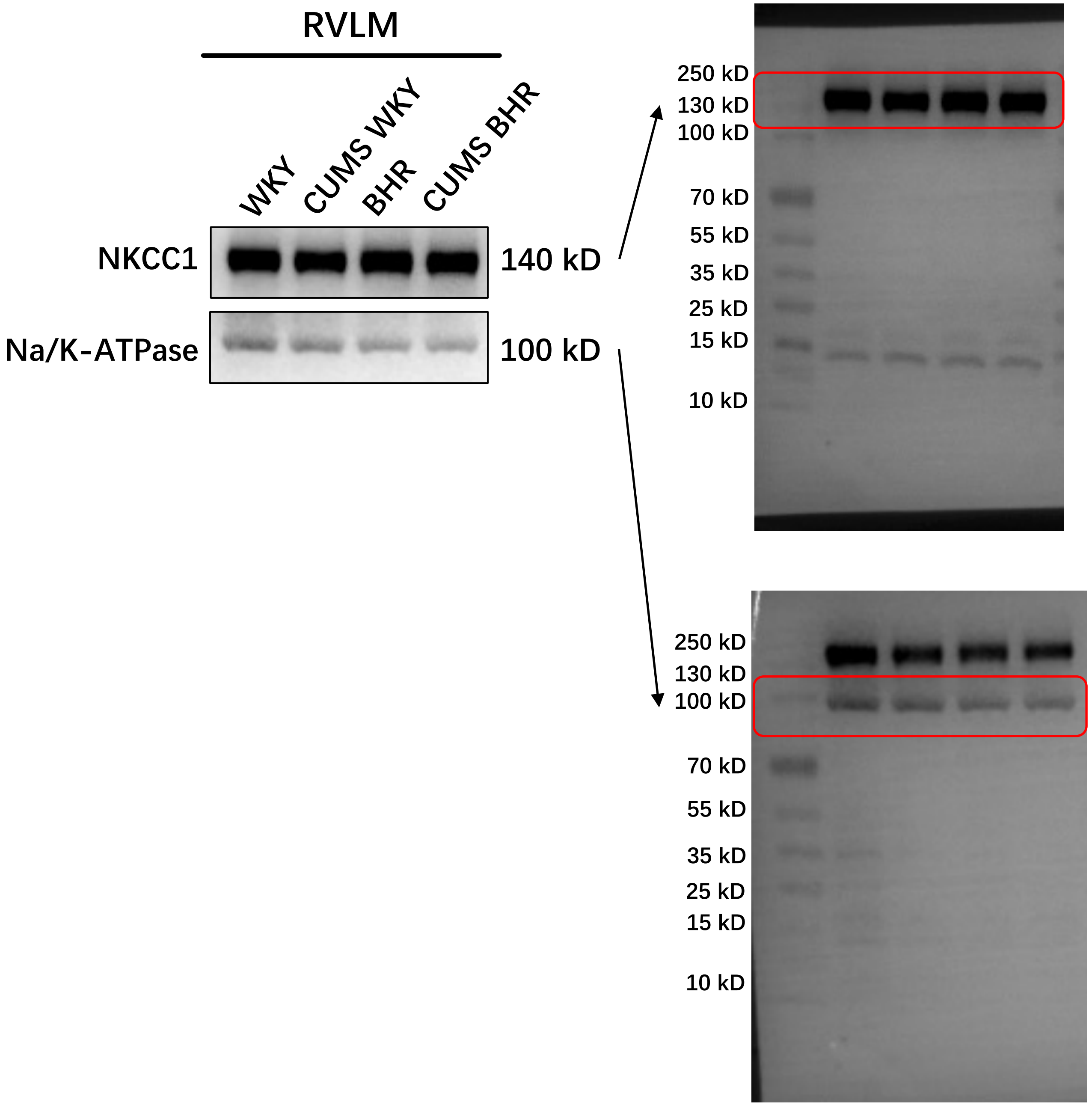

- Full unedited gels for Figure 5D

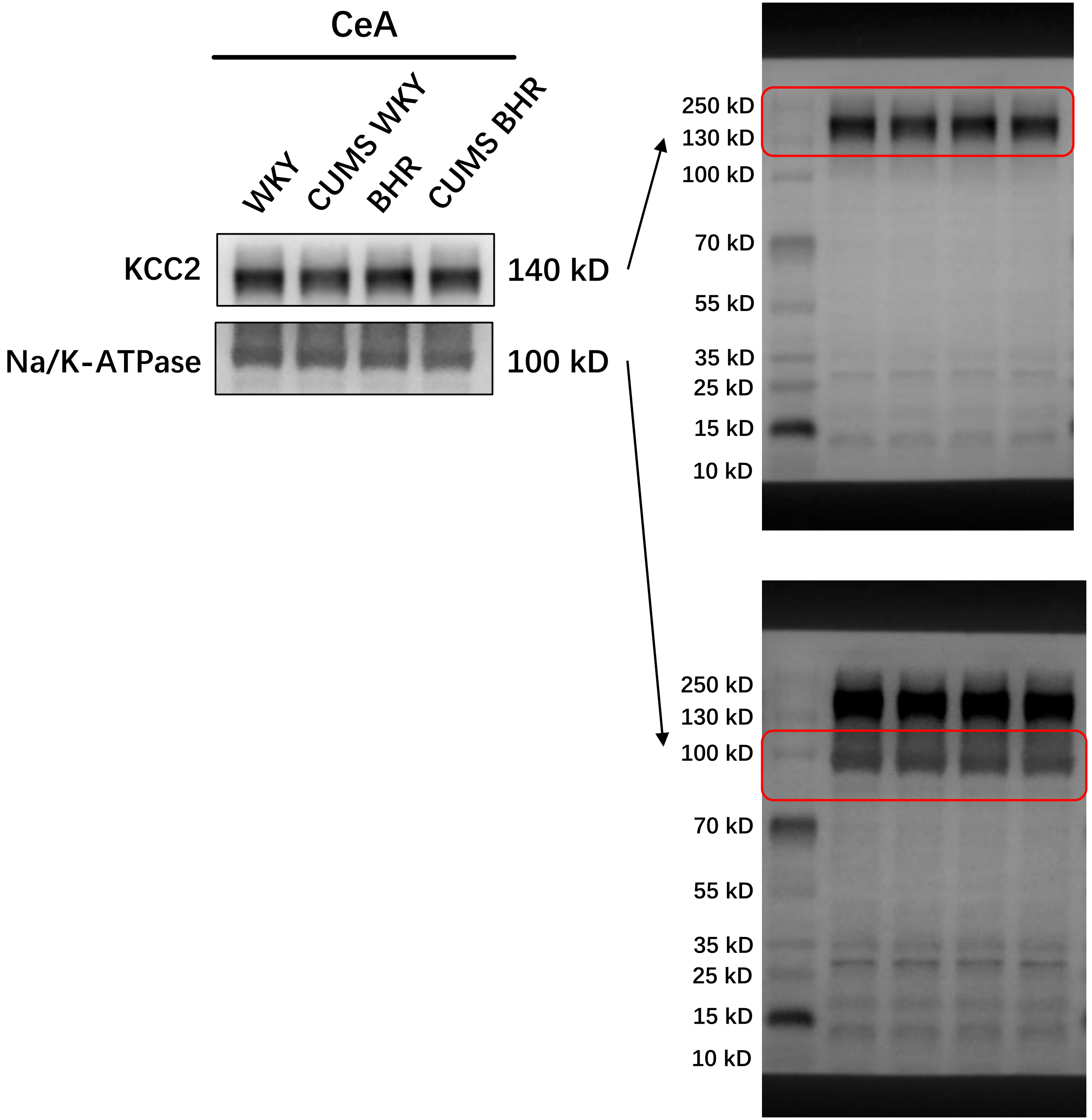

- Full unedited gels for Figure 5E

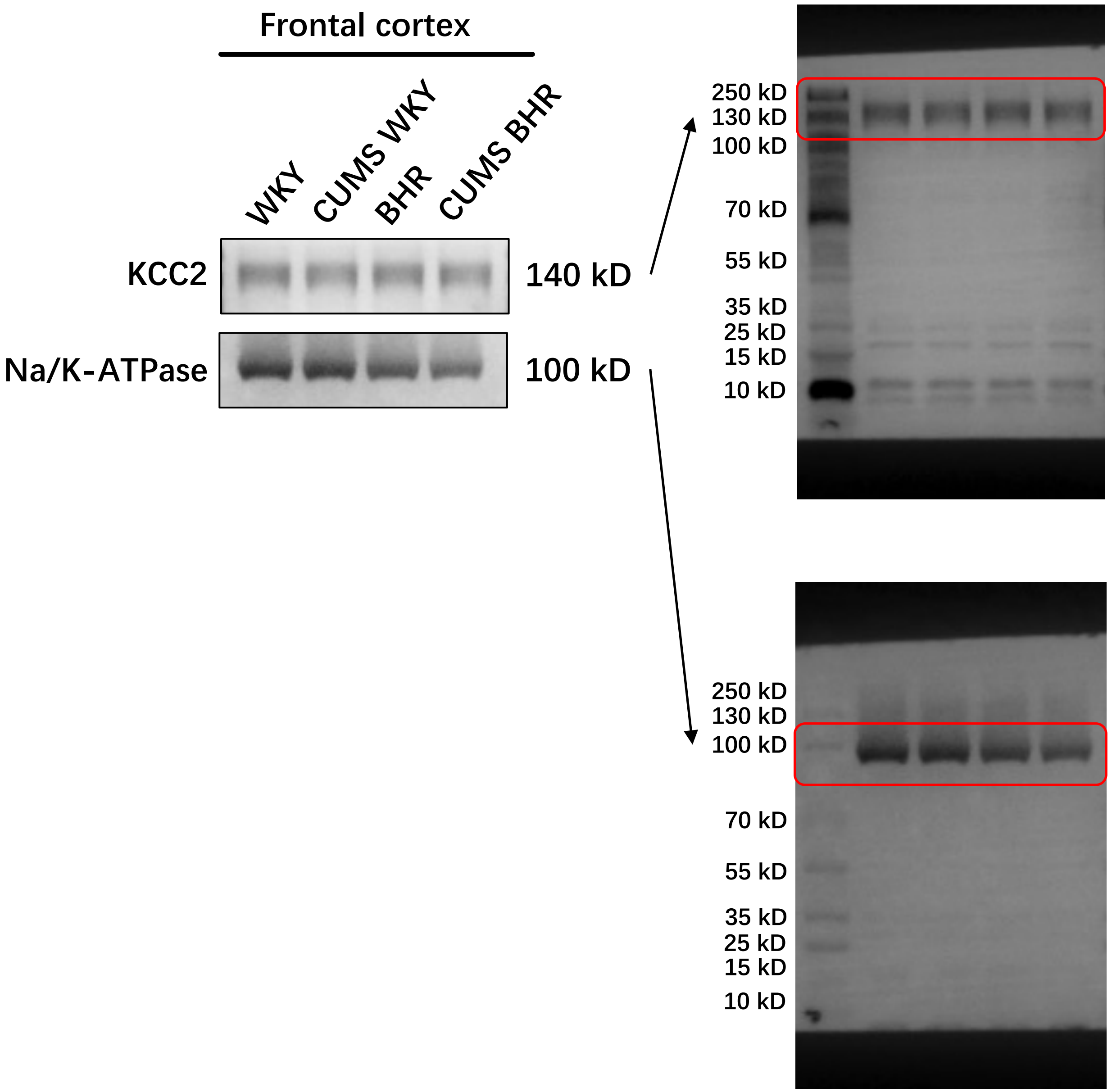

- Full unedited gels for Figure 5F

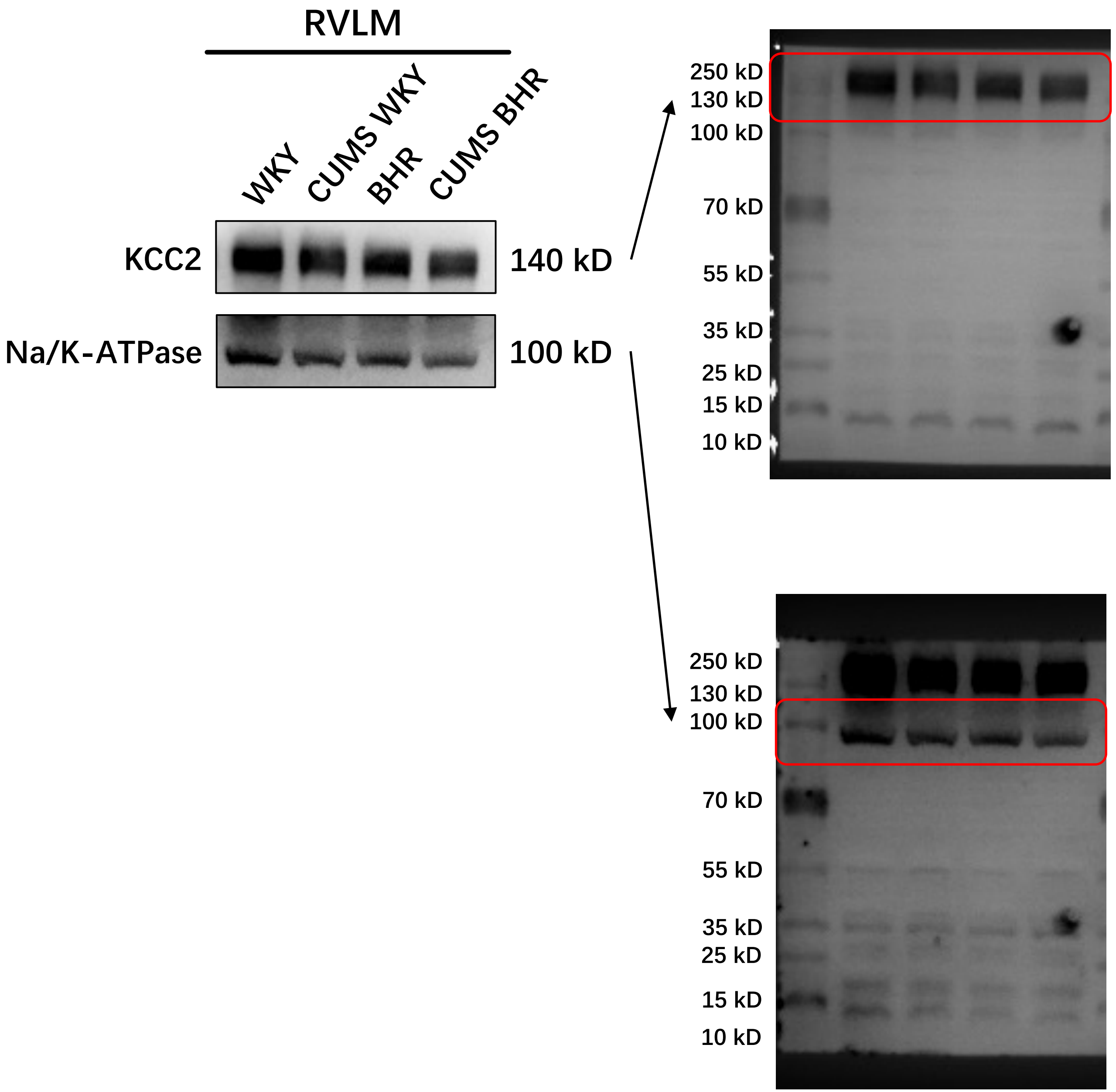

- Full unedited gels for Supplementary figure 4B

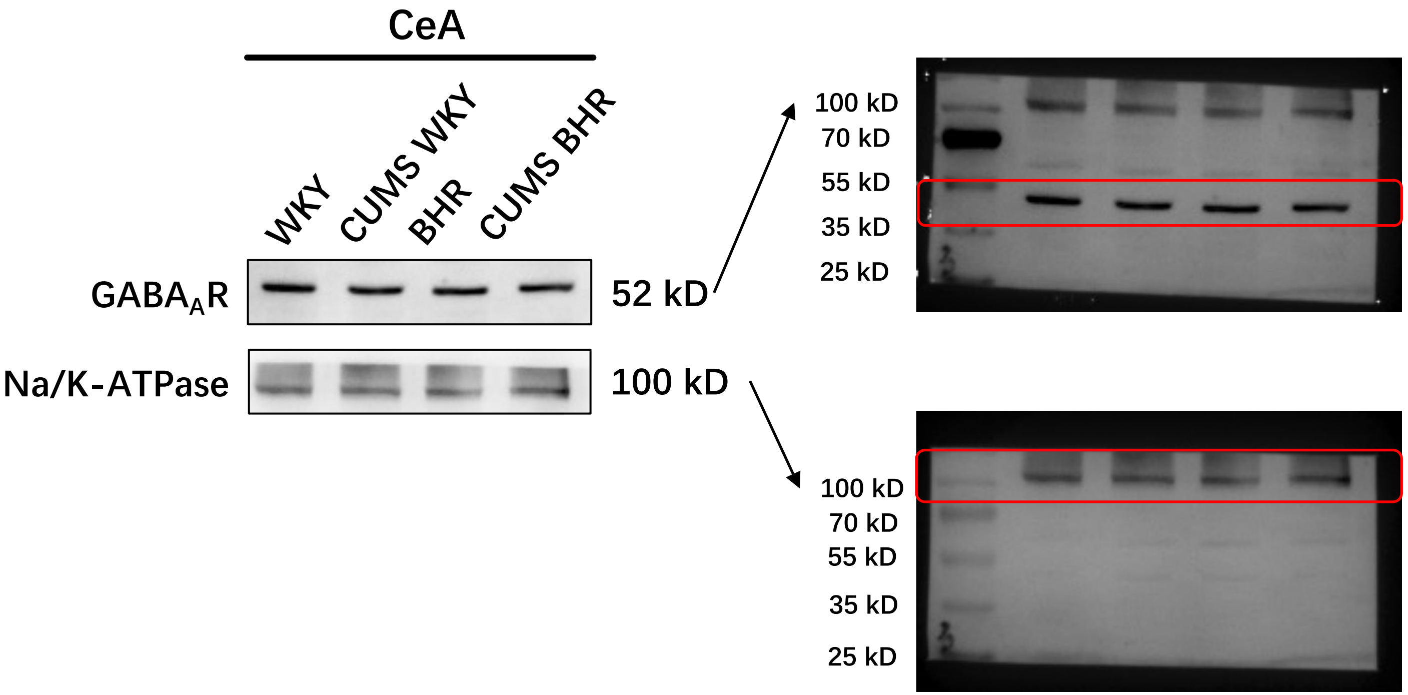

Supplement: Unedited blot and gel images [file jci-136-195536-s009.pdf]
